# Partial synchronisation of glycolytic oscillations in yeast cell populations

**DOI:** 10.1038/s41598-020-76242-8

**Published:** 2020-11-12

**Authors:** André Weber, Werner Zuschratter, Marcus J. B. Hauser

**Affiliations:** 1 Combinatorial NeuroImaging Core Facility (CNI), Leibniz Institute for Neurobiology Magdeburg, Brenneckestraße 6, 39118 Magdeburg, Germany; 2 Department of Regulation Biology, Institute of Biology, Otto-von-Guericke Universität Magdeburg, Pfälzer Straße 5, 39106 Magdeburg, Germany

**Keywords:** Biophysics, Physics, Biological physics, Statistical physics, thermodynamics and nonlinear dynamics

## Abstract

The transition between synchronized and asynchronous behaviour of immobilized yeast cells of the strain *Saccharomyces carlsbergensis* was investigated by monitoring the autofluorescence of the coenzyme NADH. In populations of intermediate cell densities the individual cells remained oscillatory, whereas on the level of the cell population both a partially synchronized and an asynchronous state were accessible for experimental studies. In the partially synchronized state, the mean oscillatory frequency was larger than that of the cells in the asynchronous state. This suggests that synchronisation occurred due to entrainment by the cells that oscillated more rapidly. This is typical for synchronisation due to phase advancement. Furthermore, the synchronisation of the frequency of the glycolytic oscillations preceded the synchronisation of their phases. However, the cells did not synchronize completely, as the distribution of the oscillatory frequencies only narrowed but did not collapse to a unique frequency. Cells belonging to spatially denser clusters showed a slightly enhanced local synchronisation during the episode of partial synchronisation. Neither the clusters nor a transition from partially synchronized glycolytic oscillations to travelling glycolytic waves did substantially affect the degree of partial synchronisation. Chimera states, i.e., the coexistence of a synchronized and an asynchronous part of the population, could not be found.

## Introduction

Synchronisation is a frequent manifestation of individual entities showing oscillatory or excitable dynamics where they adhere to a collective, common rhythm. Thus, synchronisation is a phenomenon that leads to the emergence of collective, macroscopic dynamics^1–3^. Among the abundant biological systems that show sustained oscillations^3–6^, glycolytic oscillations play a prominent role because glycolysis is the fundamental pathway in the energy metabolism, which is conserved from bacteria to humans. For instance, glycolytic oscillations have been observed in the bacterium *Escherichia coli*^7^, the yeasts *Saccharomyces carlsbergensis*^8–13^ and *Saccharomyces cerevisiae*^14–21^, in murine fat cells^22^ and $\beta$-cells in islets of Langerhans^23^, ventricular myocytes of rabbits^24^, as well as in cancer cells in humans^25^. Despite its ubiquity, the physiological function of glycolytic oscillations remains unknown, however, these oscillations are reported to emerge due to a trade-off between efficiency and robustness of the glycolytic pathway^26^. One possible role of glycolytic oscillations is to act as pacemakers for other physiological oscillators^23^. On the other hand, life in a community is considered to be a strategy of long-term survival for unicellular organisms such as yeast^27^. Hence, metabolic oscillations may provide an efficient mechanism for communication among individual cells of a population.

In dense populations, yeast cells synchronize their metabolic oscillations^9,12,15^ as shown in experiments where two yeast populations initially oscillated at opposite phases but the same frequency. When they were mixed, the cells synchronized their metabolism to a common rhythm after a few oscillations^11,15^. Individual cells are coupled to each other through acetaldehyde^15,28^, which is produced and released by the yeast cells. This messenger molecule then diffuses through the extracellular medium and it is taken up by other cells. When a sufficiently high concentration of this messenger is reached locally, the cells synchronize their rhythm to that of the messenger-rich extracellular medium in the immediate environment of the cell. Synchronisation of the metabolism of yeast is not only observed in stirred cell media but also in spatially-extended arrangements, for instance, in sedimented^29^ or gel-entrapped cells^30,31^.

Already early on, the collective dynamics of yeast was found to depend on cell density. At high cell densities, the individuals synchronize their metabolic oscillations, whereas collective oscillations are no longer displayed when the cell density falls below a critical threshold^16,32^. The transition between these two types of macroscopic dynamics may follow two distinct pathways. First, the transition may occur through a ’Kuramoto transition’^33,34^, where, at high cell densities, all cells oscillate with common phase and frequency, whereas in sparse populations, the individual cells continue to oscillate, however, they lose their phase (and possibly frequency) coherence. The second pathway is either called ’oscillation death’ in the field of Dynamical Systems^35^ or ’dynamical quorum sensing’ in more biological literature^36,37^. Here, the transition between collective oscillations and quiescence is caused by a simultaneous cessation (or onset) of the oscillations at the level of individual cells. In other words, here, the dynamics of the individuals is the same as that of the population.

The character of the transition between synchronous collective oscillations and quiescence on the macroscopic level depends on both, cell density and the strength of the coupling between oscillators (or cells). In fact, the phase space domains supporting Kuramoto transition and oscillation death are separated by a common phase boundary^35,38^. Both types of transitions, i.e., oscillation death and the Kuramoto transition, have been reported to occur in yeast cell populations. Whereas oscillation death was found in stirred yeast cell suspensions^36^, in suspended and stirred beads of an oscillatory reaction^37^, or even in immobilized cells^18^, early reports that individual cells remain oscillatory in unstirred cell suspensions at low cell densities^14^ have recently been confirmed in experiments using either *S. carlsbergensis* cells immobilized on coverslips^13^ or *S. cerevisiae* in microfluidic devices^20,39^.

For systems with a unimodal frequency distribution, the Kuramoto transition between synchronous and asynchronous dynamics is a second order phase transition^40^. For small numbers of oscillators (or cells), however, this transition persists but becomes blurry^41^. In the case of yeast cell populations this implies that for suitable cell densities (which we call intermediate), partial synchronisation of the cells occurs, i.e., the population is neither completely synchronized to a unique frequency and phase, nor does it oscillate in an asynchronous manner. An investigation of such partially synchronized states may shed some light into the route through which immobilized yeast cells achieve synchronisation. On the other hand, partial synchronisation may involve so-called chimera states. In a chimera, the coupling of originally identical oscillators leads to a symmetry breaking, such that (at least) a subpopulation of the oscillators is synchronized to each other, whereas the other subpopulation remains desynchronized^42–44^. In fact, chimera states have also been intensively studied in a variety of coupled oscillatory systems^45–48^, both in theory and experiments. In the latter, the individual oscillators were no longer identical, but similar to each other, due to the inevitable presence of noise.

In the present study, we investigate how populations of immobilized *S. carlsbergensis* cells achieve synchronisation by monitoring the autofluorescence of the coenzyme NADH. To this purpose, we profit from the relative longevity of both the asynchronous and the partially synchronized states in populations of intermediate density. In addition to the temporal dynamics, we study the changes in the spatial aspects of the immobilized cells during the transition between the asynchronous and the partially synchronized state. Furthermore, we investigate the evolution of the spatial coherence of the oscillations during the partially synchronized state. Finally, we tested whether the partially synchronized state supports the generation of chimera states.

## Results

The dynamics of glycolytic oscillations of yeast cells of the strain *S. carlsbergensis* depended on the cell density. While at cell densities $\rho > 0.3\%$ all cells synchronized their metabolism to a joint rhythm (Fig. S11), synchronisation could not be attained for populations of cell densities $\rho<$ 0.08% (Fig. S12). As the goal of the present paper is to study the details of partial synchronisation of the glycolytic oscillations in yeast cells, we have focused on the behaviour of populations of intermediate cell densities, i.e., 0.08% $\le \rho \le$ 0.3% that contained from (8–11) $\times$ 10$^6$ to (30–42) $\times$ 10$^6$ cells $\hbox {ml}^{-1}$. At these intermediate cell densities, the populations may present both a state where cells oscillated asynchronously (i.e., a desynchronized state) and a state where the glycolytic oscillations of the cells were partially, but not completely, synchronized.

Although experiments were run at several cell densities (ranging from $\rho = 0.01$% to 0.7%), in the following, we focus on two experiments performed at $\rho = 0.1$% and 0.3% in order to present the typical details of partial synchronisation in yeast cell populations, as well as the pathway leading to it, in a concise manner.

### Partial synchronisation of yeast cells

**Figure 1:**
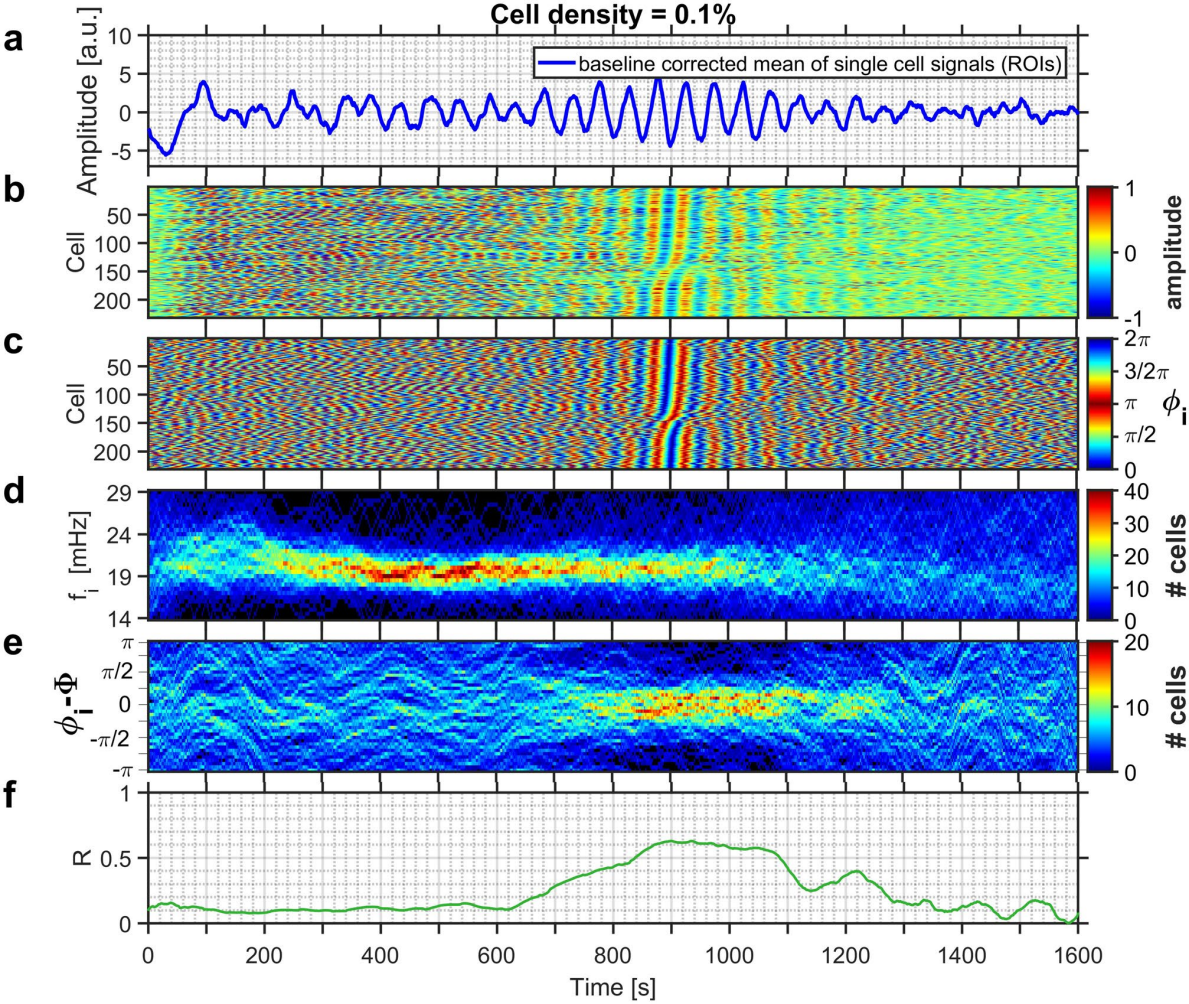
(**a**) The time-series of the collective NADH fluorescence signal for a yeast population of cell density $\rho = 0.1\%$. Partial synchronisation of intracellular oscillations occurs at 760 s $\le t \le 1100$ s. (**b**) Development of the relative amplitudes of oscillations of each cell, and (**c**) of their phases. In (**b**) and (**c**) the cells are sorted according to their phases at time $t = 900$ s. (**d**) Evolution of the distribution of instantaneous frequencies $f_i$ of the cells, and (**e**) of the distribution of the phase difference $\Delta \phi _i$ between the phase $\phi _i$ of each individual cell to that of the average phase $\Phi$ of all cells of the population. (**f**) Time dependence of the order parameter *R*. The field of view had a diameter of 169 $\mu$m, and hosted 232 cells. Glucose was added to the cell suspension at $t = -\,158$ s.

**Figure 2:**
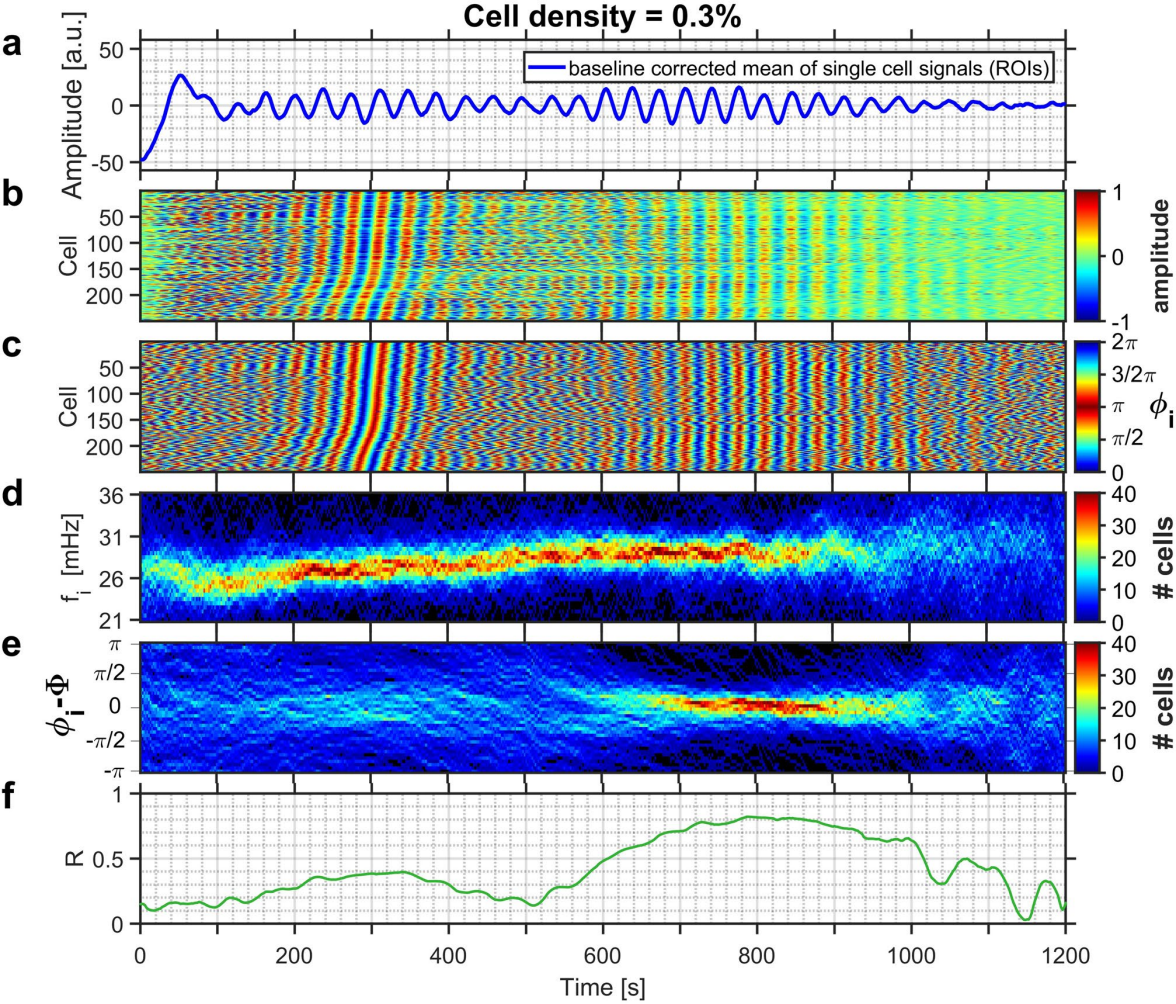
(**a**) The time-series of the collective NADH fluorescence signal for a yeast population of cell density $\rho = 0.3\%$. Partial synchronisation of intracellular oscillations occurs at 580 s $\le t \le 1080$ s. (**b**) Development of the relative amplitudes of oscillations of each cell, and (**c**) of their phases. In (**b**) and (**c**) the cells are sorted according to their phases at time $t = 300$ s. (**d**) Evolution of the distribution of instantaneous frequencies $f_i$ of the cells, and (**e**) of the distribution of the phase difference $\Delta \phi _i$ between the phase $\phi _i$ of each individual cell to that of the average phase $\Phi$ of all cells of the population. (**f**) Time dependence of the order parameter *R*. The field of view had a size of $85\,\times \,85$ $\mu$m$^2$ and hosted 251 cells. Glucose was added to the cell suspension at $t = -\,210$ s.

The addition of an aliquot of glucose to the medium containing starved yeast induced intracellular glycolytic oscillations. The global dynamics of the entire population as well as that of the individual cells are displayed in Figs. 1 and 2 for experiments run at cell densities $\rho = 0.1\%$ and $\rho = 0.3\%$, respectively. At the level of the population, the fluorescence signal began to show irregular, aperiodic low-amplitude oscillations, which eventually increased in amplitude at $t \approx 760$ s in Fig. 1a and $t \approx 580$ s in Fig. 2a. These pronounced and rather regular oscillations lasted until $t \approx 1100$ s in Fig. 1 and $t \approx 1020$ s in Fig. 2, after which the oscillations dampened substantially (Figs. 1a, 2a). Eventually the oscillations ceased completely, as the experiments were conducted under batch conditions and the nutrient glucose was only added once at the begin of the experiment.

The degree of synchronisation in the entire population is given by the order parameter *R*, which increased to values $R > 0.40$, but always remained below $R < 0.85$ (Figs. 1f, 2f), indicating that the cells became partially synchronized. The rationale for choosing these boundaries for *R* reflects, on the one hand, the low number of cells in the field of view. The lower limit made sure that spurious, but short-lasting coincidental oscillations were not counted as being synchronized. On the other hand, the upper limit still ensured that the population was not yet completely synchronized. Partial synchronisation was observed in the interval lasting from $t \approx 760$ s to $t \approx 1100$ s in the less dense population (i.e., $\rho = 0.1\%$) shown in Fig. 1) and from 580 s to $t \approx 1080$ s in the denser population (of $\rho = 0.3\%$) displayed in Fig. 2.

After addition of glucose, the individual cells began to develop glycolytic oscillations, whose frequencies were broadly distributed. After this induction period, which usually lasted for a few oscillation periods, the oscillations attained pronounced amplitudes and the oscillation frequencies were steadied. This led to a narrowing of their frequency distribution. The induction period then gave way to a regime of asynchronous oscillations, where the cells oscillated at individual phases (e.g., at 200 s $\le t \le 760$ s in Fig. 1; 100 s $\le t \le 580$ s in Fig. 2). As the asynchronous dynamics proceeded, the intracellular oscillations got in tune, eventually leading to a synchronisation of the glycolytic oscillations in the individual cells (after $t > 760$ s in Fig. 1b,c, and after $t > 580$ s in Fig. 2b,c). Interestingly, in populations of intermediate cell density, the synchronisation among the cells set in when the oscillation amplitudes of the individual cells had already begun to decay (Figs. 1b, 2b, S3, and S6).

**Figure 3:**
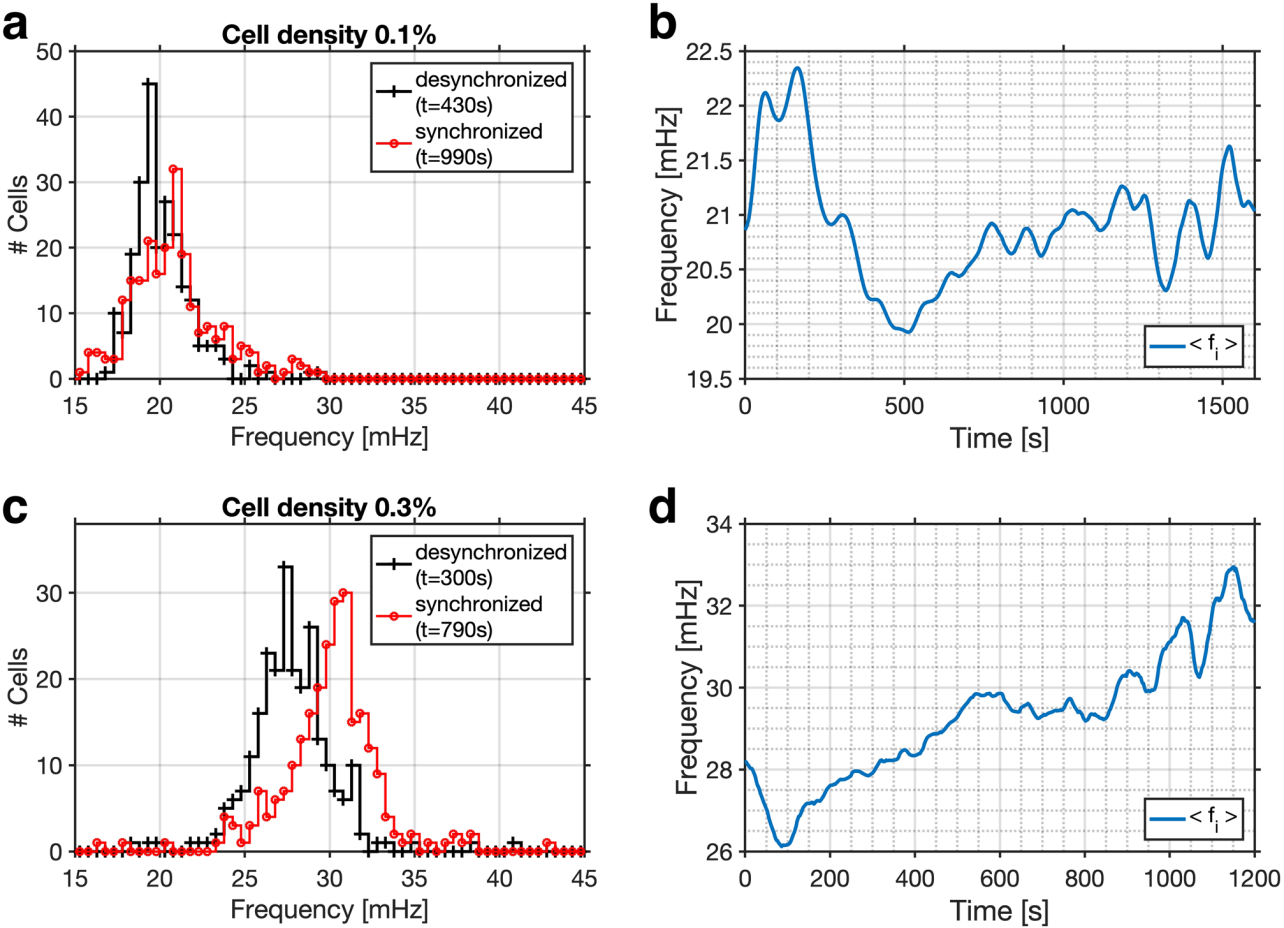
Evolution of the instantaneous frequencies. Upon partial synchronisation, the frequency distribution is shifted towards higher frequencies. (**a, c**) Histograms of the instantaneous frequencies of the oscillations of individual cells during asynchronous (desynchronized; black line) and partially synchronized (red line) oscillations. The instantaneous frequencies were binned into intervals of $\delta f = 0.5$ mHz. (**b, d**) Temporal evolution of the mean of the frequency distribution. In a population of $\rho = 0.1\%$ (shown in Fig. 1), (**a**) the frequency distribution is shifted to slightly higher frequencies and its mean increases from (**a**) 20.0 to 21.0 mHz (at $t = 430$ s and $t = 990$ s, respectively). (**b**) Partial synchronisation is achieved as the mean of the frequency distribution reaches and remains on a plateau (from $t \approx 750$ to 1100 s). In a population of $\rho = 0.3\%$ (shown in Fig. 2), (**c**) the frequency distribution is shifted to slightly higher frequencies and its mean increases from (**a**) 27.5 to 30.0 mHz (at $t = 300$ s and $t = 790$ s, respectively). (**d**) Partial synchronisation is achieved as the mean of the frequency distribution reaches and remains on a plateau (from $t \approx 550$ to 950 s).

The evolution of the distributions of the frequencies $f_i$ (Figs. 1d, 2d, 3) and of the relative phases $\Delta \phi _i$ (Figs. 1e, 2e) reveal the pathway that leads to partial synchronisation of yeast cells. During the regime of asynchronous oscillations, the frequency distribution narrowed slightly as the cells began to synchronize (at $t \approx 400$ s and $\approx 240$ s in Figs. 1d, 2d). In addition, a shift of the mean of the frequency distribution to higher values is correlated to the phase synchronisation of the cells. In the experiment shown in Fig. 1 this occurred at $t \approx 700$ s, where the mean frequency increased from 20.0 to 21.0 mHz (this corresponded to a shortening of the mean oscillation period from $T = 50.0$ to $T = 47.6$ s; see also Fig. 3a,c), whereas in the experiment in Fig. 2d the mean frequency increased from 27.5 to 30.0 mHz (corresponding to a decrease in the mean period from $T = 36.4$ s to $T = 33.3$ s; see also Fig. 3b,d). The shape of the frequency distribution depended on the dynamic state of the population: While the histogram of desynchronized cells is either symmetric or slightly skewed to higher frequencies, the histogram of the distribution of partially synchronized cells is skewed to the other side, namely to shorter frequencies (Fig. 3).

The narrowing of the distribution of the relative phases of the oscillations $\Delta \phi _i$ (Fig. 1e) was substantially delayed when compared to the narrowing of the distribution of the frequencies $f_i$ (Fig. 1d): The distribution of the relative phases narrowed and sharpened only after the mean of the distribution of the frequencies had attained a higher and stationary value. For the population of cell density $\rho = 0.1\%$ shown in Fig. 1d,e the increase in the mean frequency was reached at $t \approx 700$ s while phase synchronisation was only attained at $t \approx 800$ s. A similar behaviour was also observed in denser populations (e.g., at $\rho = 0.3$%), where the shift in the mean frequency occurred at $t \approx 560$ s, whereas it took an additional $\approx 100$ s to synchronize the phases (Fig. 2d,e). These results demonstrate that frequency synchronisation preceded phase synchronisation (Figs. 1d,e, 2d,e).

**Figure 4:**
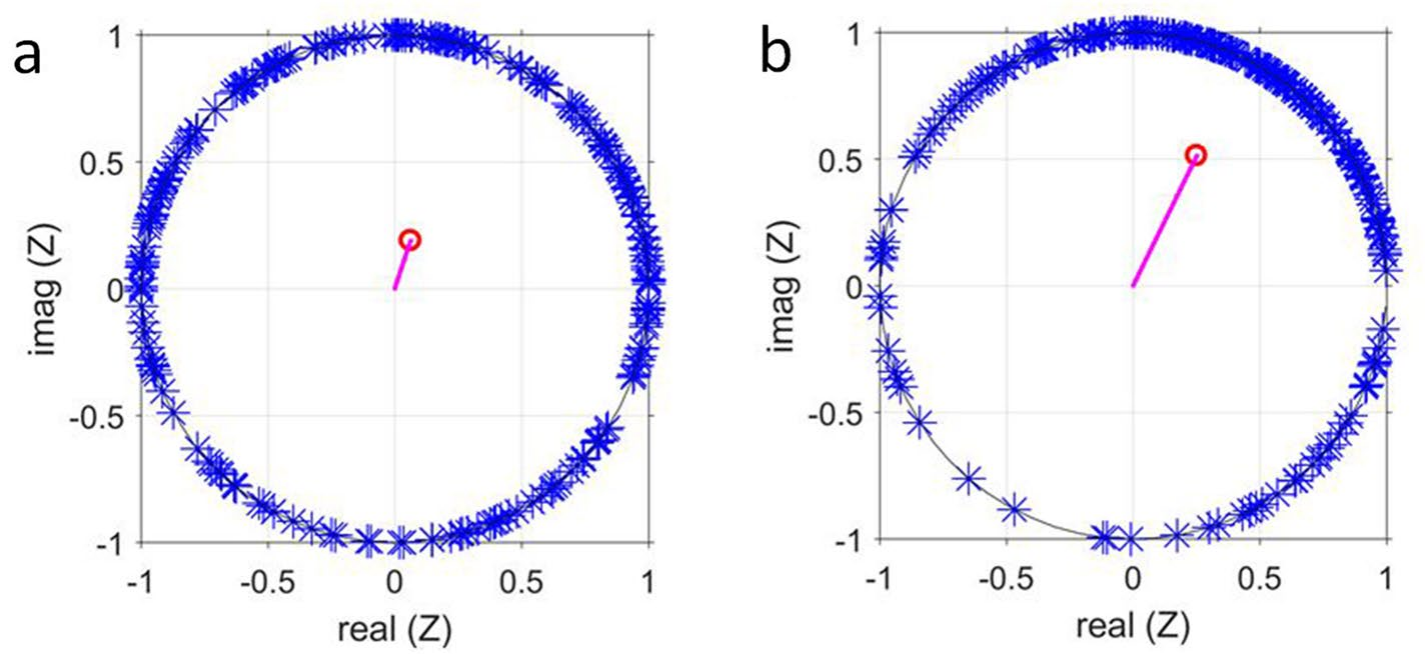
The phases $\phi _i$ of the glycolytic oscillations of every cell of a population of cell density $\rho = 0.1\%$ (Fig. 1) are plotted on the unit circle (**a**) during the regime of asynchronous oscillations at $t = 518$ s and (**b**) during partial synchronisation at $t = 904$ s. The position of the phase of each cell is marked by an asterisk. The vector starting at the origin points to the value of the average phase $\Phi$, and the magnitude of the vector corresponds to the value of the order parameter *R* (which is (**a**) $R = 0.14$ and (**b**) $R = 0.61$, respectively).

Further evidence for the partial nature of the synchronisation is provided by the analysis of the dynamics at points in time that belong to different dynamic regimes, namely one during asynchronous oscillations and the other during partial synchronisation (e.g., at $t = 518$ s and $t = 904$ s, respectively, in Fig. 4, which shows data of the sparse population of $\rho = 0.1\%$. Data for $\rho = 0.3\%$ are provided in Fig. S7). Phase plots, where the phases $\phi _i$ of the cells were plotted on the unit circle, demonstrate that during the regime of asynchronous oscillations the phases of the individual cells were well spread over the unit circle (Fig. 4a), whereas the order parameter was low. The partial nature of the synchronisation is illustrated by the phase plot, where at $t = 904$ s, most cells occupied the upper right quadrant of the unit circle; however, there still was a considerable number of cells, whose phases $\phi _i$ deviated from the average phase $\Phi$ (Fig. 4b). Here, the order parameter *R* was higher, but still far from unity (as required for complete synchronisation). A similar situation was found for data obtained at all intermediate cell densities (i.e., $0.08\% \le \rho \le 0.3\%$).

### Spatial aspects of partial synchronisation

**Figure 5:**
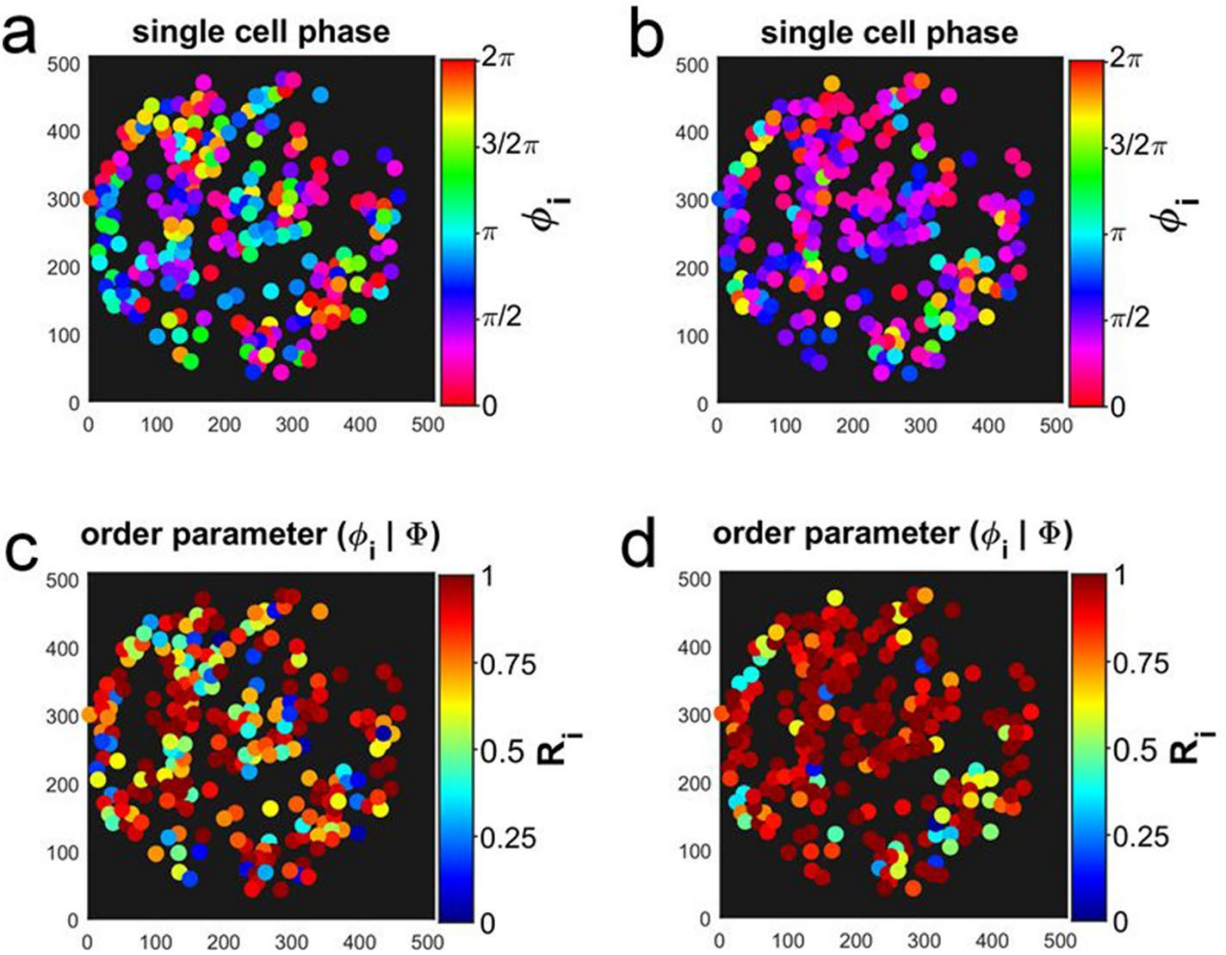
Spatial partition of the phases $\phi _i$ of the glycolytic oscillations of each cell (**a**) during asynchronous oscillations ($t = 518$ s) and (**b**) during the partially synchronized state (at $t = 904$ s). Spatial partition of the relative order parameter $R_i$ (Eq. 6) (**c**) during the regime of asynchronous oscillations ($t = 518$ s) and (**d**) during the partially synchronized state (at $t = 904$ s). Data from the experiment at cell density $\rho = 0.1\%$ shown in Fig. 1. The field of view had a diameter of 169 $\mu$m and it was binned into 512 $\times$ 512 pixels. Each pixel had a resolution of 0.33 $\mu$m $\times$ 0.33 $\mu$m.

When investigating immobilized cell populations of intermediate cell densities, the spatial organization of the dynamics within the population must also be taken into account. To this purpose, the spatial distributions of the phases $\phi _i$ and the relative order parameter $R_i$ of the immobilized cells were calculated and mapped onto the positions of the immobilized cells (Fig. 5). For each point in time, we have also calculated the oscillation amplitude, phase $\phi _i$, instantaneous frequency $f_i$, oscillation period $T_i$ of each cell, as well as the relative order parameter $R_i$ and the phase shift $\Delta \phi _i$ of each cell with respect to the main phase $\Phi$. In the Supplementary Information, we provide a plot and a video where the obtained values of these parameters were mapped onto the positions of their respective immobilized cells (Fig. S1 and Video S1; Fig. S4 and Video S4).

The spatial distribution of the phases $\phi _i$ and the relative order parameter $R_i$ of the immobilized cells were plotted for characteristic points in time in Fig. 5 for the time series of $\rho = 0.1\%$ shown in Fig. 1 (and Fig. S8 for $\rho = 0.3\%$). It is evident that during the episode of asynchronous oscillations, where the phases of the individual cells were rather incoherent, there were neither spatial correlations of the position of the cell with its phase $\phi _i$ (Fig. 5a) nor with its relative order parameter $R_i$ (Fig. 5c). During partial synchronisation, the phases $\phi _i$ of the cells became more coherent in space. This is demonstrated in Fig. 5b, which depicts the situation at $t = 904$ s, where most cells oscillated at a phase between 0 and 1/2 $\pi$ (which corresponds to the upper right quadrant in Fig. 4b), especially in the upper left part of the field of view of Fig. 5b. Accordingly, the relative order parameter $R_i$ of most of the cells has risen close to unity and their dynamics also became more coherent in space. However, a strict correlation between $\phi _i$, $R_i$, and the position of cell *i* was not yet reached, since there still were cells with deviating values of $R_i$ dispersed among those cells, whose values of $\phi _i$ and $R_i$ were rather similar, i.e., synchronized (Fig. 5b,d).

**Figure 6:**
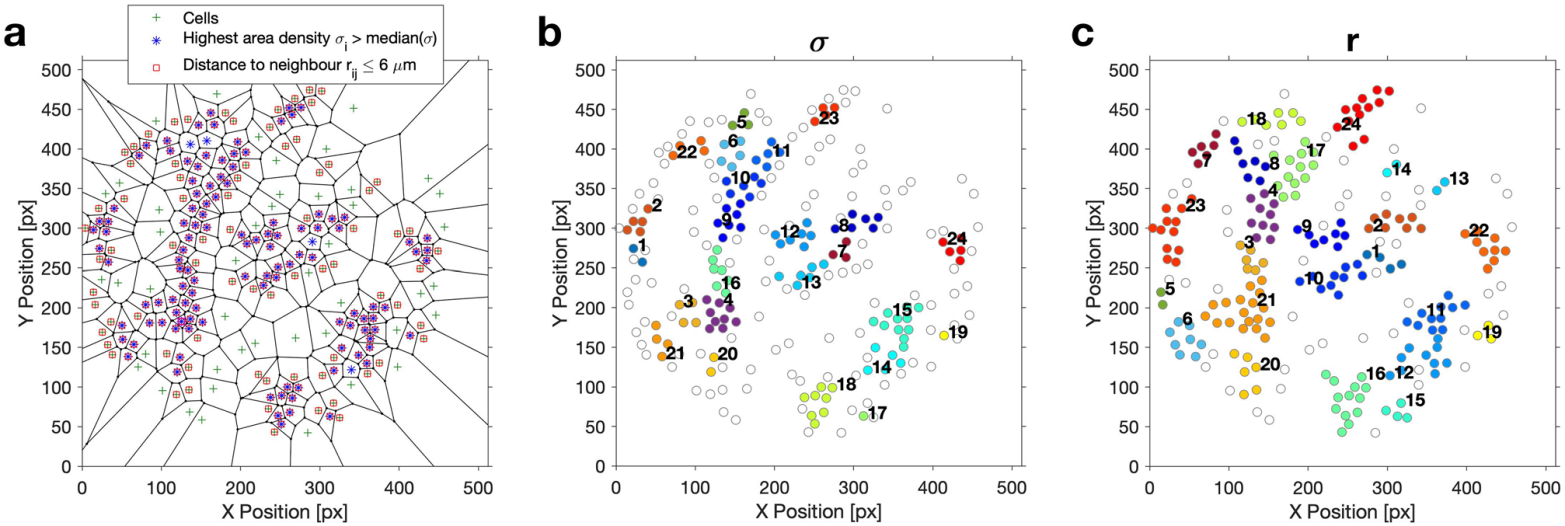
Voronoi tessellation and segmentation of the cells into spatial clusters. Cell density: 0.1%. (**a**) In the Voronoi diagram, the positions of the centres of mass of the cells are indicated by green crosses; the cells displaying the highest relative cell area densities $\sigma _i > median(\sigma _i)= 2.53$ and those with the shortest intercellular distances $r_{ij} \le 6$ $\mu$m are indicated by blue asterisks and red squares, respectively. (**b**) Clusters obtained due to segmentation with respect to the (highest) relative cell area density $\sigma _i$ and (**c**) with respect to the (shortest) intercellular distances $r_{ij}$. The field of view had a diameter of 169 $\mu$m and it was binned into $512\,\times \,512$ pixels. Each pixel had a resolution of 0.33 $\mu$m $\times$ 0.33 $\mu$m.

**Figure 7:**
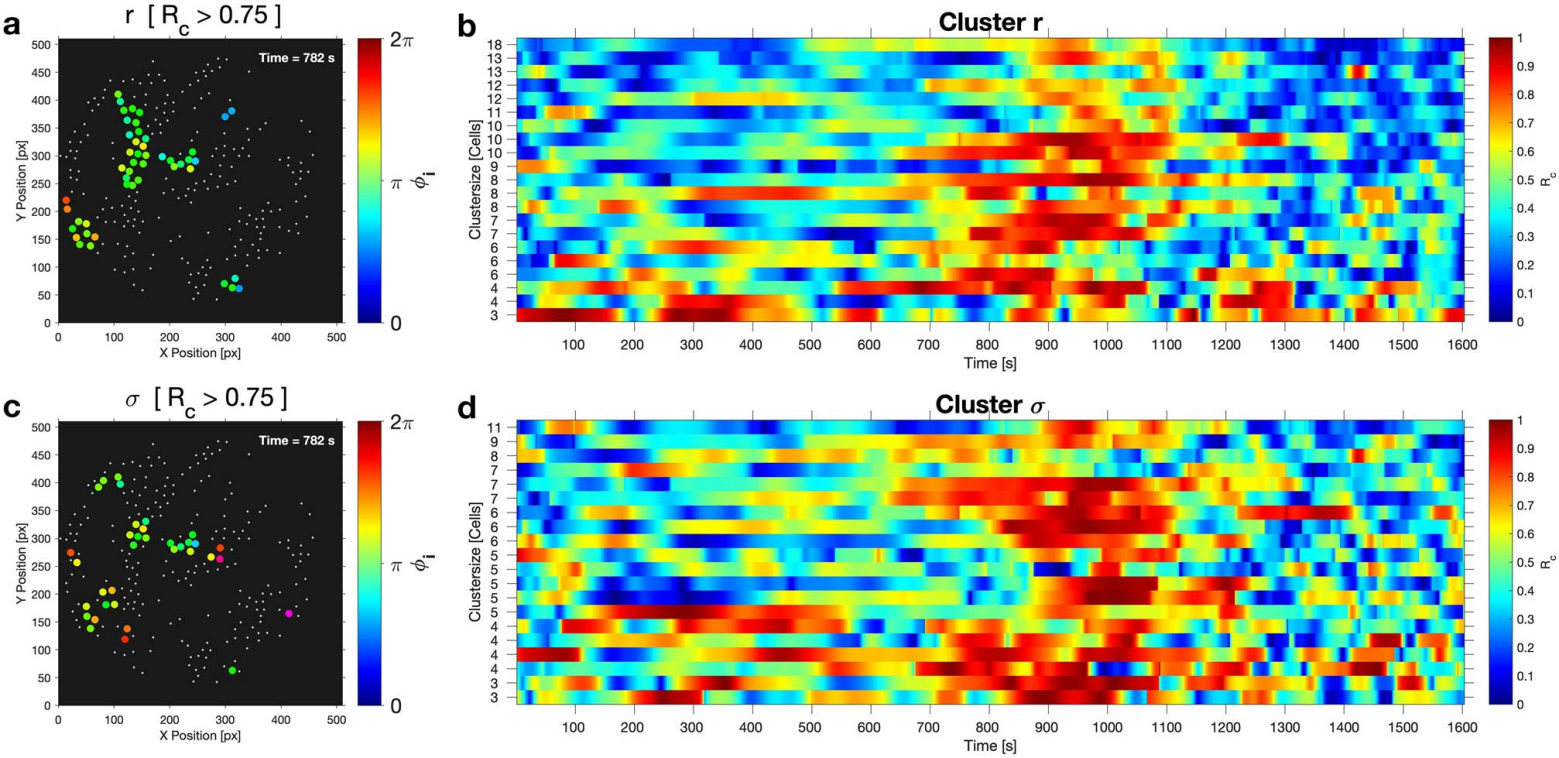
Maps of cells in clusters with enhanced synchronisation and cluster order parameter $R_c$ for each cluster of the cell population of density $\rho = 0.1\%$ (shown in Fig. 1). (**a, c**) Maps of clusters with enhanced synchrony during partial synchronisation. When the cluster order parameter exceed the threshold of $R_c \ge 0.75$, the phases $\phi _i$ of the cells were mapped onto their positions, else the cells are indicated by points. Clusters obtained by segmentation with respect (**a**) to $r_{ij}$ and (**c**) to $\sigma _i$. Video S2 compiles the time evolution of the dynamics in the spatial clusters. Persistence of the enhanced synchrony in the clusters obtained by segmentation (**b**) with respect to $r_{ij}$ and (**d**) to $\sigma _i$.

**Figure 8:**
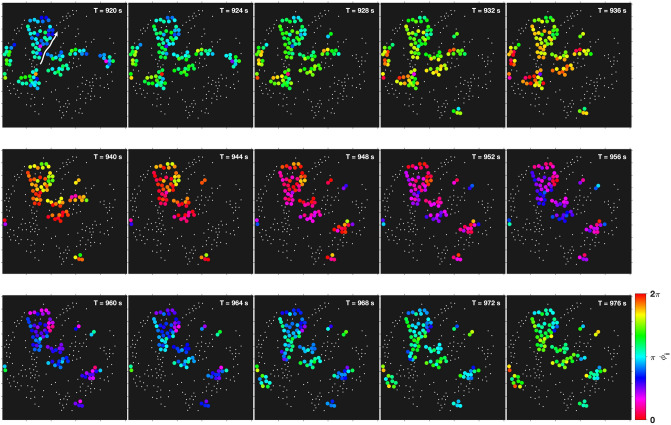
Visualization of a glycolytic wave travelling in the cell population (see also Video S2). The wave propagates almost diagonally through the field of view, from the bottom left to the top right. Cells that lie closer to the bottom left are always slightly phase-advanced with respect to any other cell that is located closer to the top right. The phase gradient is also well developed in the central cluster (that reaches roughly from the top left to the centre of the field of view). The arrow in the top left panel is a guideline for the eye, indicating the direction of propagation of the glycolytic wave. The snapshots were taken at intervals of $\Delta t = 4$ s, and the clusters obtained by segmenting with respect to the intercellular distance $r_{ij}$. The visualized cells displayed a local cluster order parameter of $R_c \ge 0.75$. The field of view had a diameter of 169 $\mu$m and it was binned into 512 $\times$ 512 pixels. Each pixel had a resolution of 0.33 $\mu$m $\times$ 0.33 $\mu$m.

On a local scale, some cells were more densely packed than on average, thus forming spatial clusters, as determined after a Voronoi tessellation of the area occupied by each cell on the coverslip and a segmentation with respect to either the relative cell area density $\sigma _i$ or the distance $r_{ij}$ between adjacent cells (Figs. 6 and S9). Note that the shape and sizes of the clusters differed slightly in dependence on the variable used for thresholding. We have investigated how the cells in a spatial cluster were synchronized among each other by calculating the cluster order parameter $R_c(t)$ of each of the identified clusters. As we were interested in highly synchronous behaviour of the cells in a cluster, we considered cells as well-synchronized if the threshold value for the cluster order parameter was exceeded. For instance, for the sparse population of $\rho = 0.1\%$ shown in Fig. 1, we chose $R_c \ge 0.75$ as threshold. Note that this threshold value for $R_c$ is higher than the (global) order parameter $R(t) \approx 0.6$ obtained when considering all cells during partial synchronisation. In analogy to Fig. 5, we have constructed maps where we have plotted the phases $\phi _i$ of those cells belonging to a cluster, once the threshold of cluster order parameter $R_c$ was exceeded, else the cells were only represented by a point (Figs. 7a,c, 8, and Video S2). These maps reveal that during partial synchronisation, the cells in some of the spatial clusters showed an enhanced synchronisation (as $R_c(t) > R(t)$). However, the enhanced synchronisation in the clusters lasted for some oscillation periods before losing the high coherence (i.e., $R_c(t)$ fell below the threshold), while, in turn, other clusters have become more coherent (Fig. 7b,d). The persistence times of enhanced synchrony in clusters varied form cluster to cluster, but they were always shorter than the episode of partial synchronisation. This is illustrated in Fig. 7 (and Fig. S10), where we compile the persistence time of the enhanced synchrony of the clusters.

During partial synchronisation, generally, more than one spatial cluster showed enhanced synchronisation of their cells. Although the mean phase of the glycolytic oscillations in a cluster differed from cluster to cluster, the phase difference among the clusters remained always moderate (within $\approx \frac{1}{2} \pi$), as seen from Figs. 7, 8 and Video S2. This means that partial synchronisation did not arise from a situation where the cells in the clusters are highly synchronized among each other, but are considerably phase-shifted with respect to any other cluster in the field of view.

The dynamics in the spatial clusters further revealed that transitions from synchronized oscillations to travelling glycolytic waves may take place. While in the sparse population (of $\rho = 0.1\%$, Fig. 1) partial synchronisation begun with a fairly synchronized oscillatory behaviour of the cells, propagating glycolytic waves emerged at $t \approx 900$ s. These waves rapidly travelled across the field of view as documented in the snapshots collected in Fig. 8 and in Video S2, where the glycolytic wave propagated through the field of view from the bottom left to the top right. The transition from oscillations to travelling waves is, however, not always observed in the experiments, as illustrated in Video S5 (for the experiment at $\rho = 0.3\%$, Fig. 2), which remained oscillatory until oscillations decayed and eventually ceased.

### Existence of chimera states?

Chimera states are a wide-spread phenomenon that occurs in partially synchronized systems and which have received considerable scientific attention during the last two decades. Chimera states arise from a symmetry breaking in a system of originally similar oscillators. Chimera show the coexistence of (at least) a subpopulation of oscillators that are synchronized among each other with another subpopulation that is neither synchronized with the first one nor within itself. In particular, in chimeras, the synchronized oscillators remain synchronized as long as the chimera exists.

**Figure 9:**
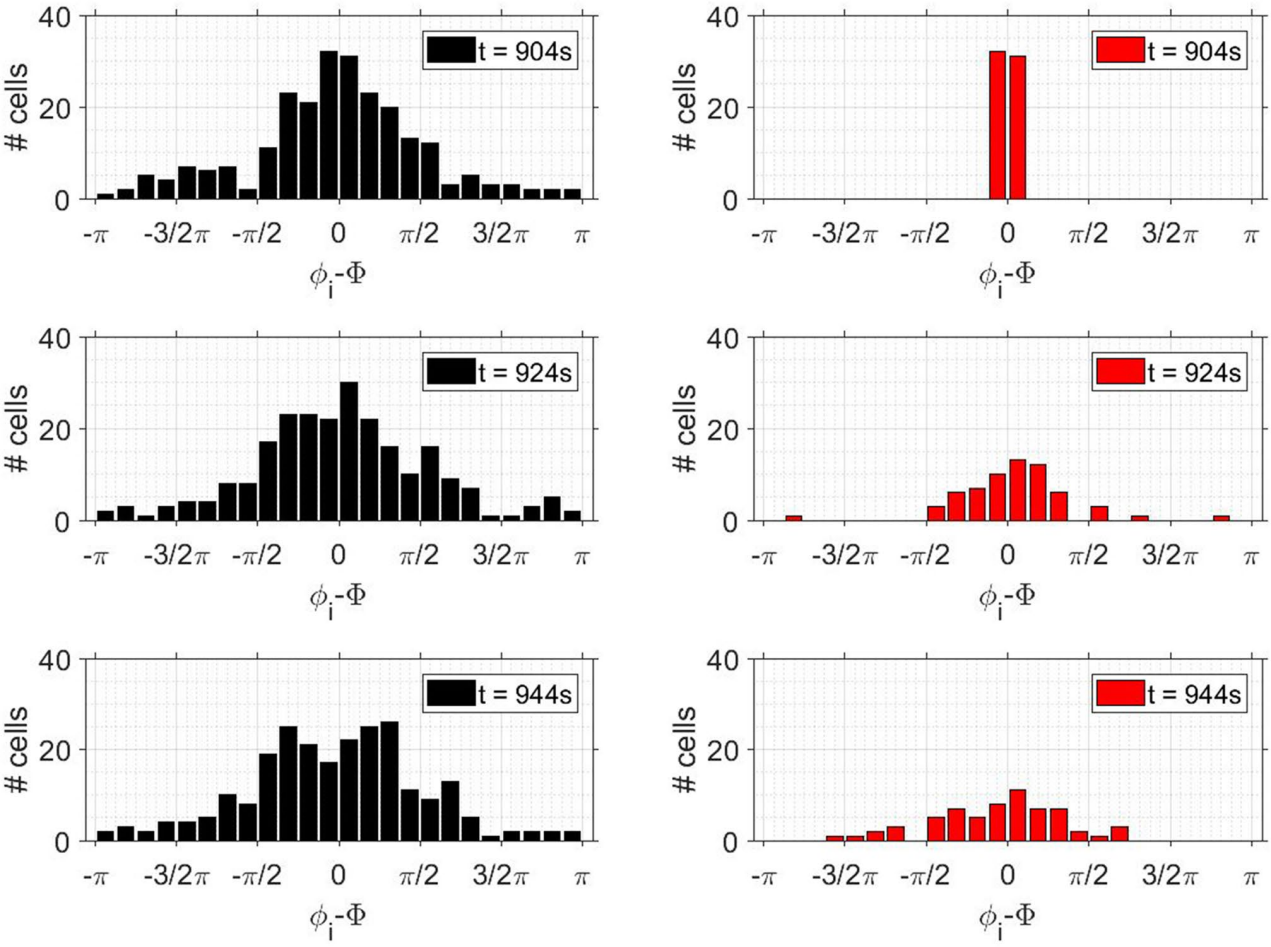
Test for chimera states. *Left row:* Distribution of the relative phases $\Delta \phi _i$ of the 232 individual cells at $t = 904, 924$, and 944 s in a population of cell density $\rho = 0.1\%$. *Right row:* Only the 103 cells that are well synchronized at $t = 904$ s are considered (i.e., cells whose phases laid within $- \frac{1}{12} \pi \le \Delta \phi _i \le \frac{1}{12} \pi$); all others are disregarded. The temporal evolution of the phase distribution of these cells that are well-synchronized at $t = 904$ s broadens rapidly, thus showing a rapid dephasing of this originally well-synchronized cells.

To test whether chimeras occurred during partial synchronisation of yeast cells, we studied the temporal behaviour of the distribution of the relative phases $\Delta \phi _i$ at different times during partial synchronisation (and in several experiments; not shown). At an instant when the order parameter *R* was high (e.g., at $t = 904$ s in Fig. 1), we focused only on the highly synchronized cells, i.e., those oscillating in the interval $- \frac{1}{12} \pi \le \Delta \phi _i \le \frac{1}{12} \pi$. In other words, we only considered the dynamics of those cells that were well-synchronized at a given point in time (e.g., at $t = 904$ s) and we neglected all cells that oscillated at other phases. This corresponds to cutting off the tails of the phase distribution, as shown in Fig. 9, where we plotted the temporal dynamics of both the entire distribution of the relative phases ($\Delta \phi _i$) and that of the central portion of this distribution.

The evolution of the phase distribution of the cells that were well synchronized at $t = 904$ s showed that this distribution broadened rapidly. More precisely, the tails of the distribution of $\Delta \phi _i$ were regenerated within about one oscillation period, leading to a fast dephasing of the originally well-synchronized cells (Fig. 9). This means that there was no subpopulation that remained synchronized during the episode of partial synchronisation. Since such a subpopulation is required for a chimera state, we do not observe the formation of chimera states in populations of partially synchronized yeast cells.

## Discussion

In yeast cell populations of intermediate cell densities, two different dynamic states are accessible for experimental investigation, namely an asynchronous (desynchronized) state where all cells oscillate with individual frequencies and a state of partial synchronisation. In the latter, the cells entrain their glycolytic oscillations, however, their oscillations fail to collapse on a unique frequency and a unique phase that is common to all cells. These two states are available for experimental studies, since the transition from desynchronisation to partial synchronisation takes a sufficiently long time. Thus, the pathway by which yeast cells synchronize can be addressed.

Shortly after addition of an aliquot of glucose, the yeast cells started intracellular glycolytic oscillations, however each cell did so at its individual frequency and phase. After a narrowing of the frequency distribution, a regime of asynchronous oscillations developed which eventually gave way to a partially synchronized state. From the evolution of the distributions of the oscillation frequencies and (relative) phases (Figs. 1d,e, 2d,e), it became evident that the synchronisation of the frequencies as well as an increase of the mean frequency preceded the synchronisation of the relative phases ($\Delta \phi _i$). The shift of the frequency distribution advanced the phase synchronisation by about the duration of one to two oscillatory cycles ($\approx 100$ s). However, the synchronisation among the glycolytic oscillations in the cells was only partial, i.e., not all cells oscillated with a unique phase and frequency.

The partial synchronisation of the cellular oscillations was achieved due to a characteristic transition in the frequency distribution, namely the shift of the mean to a higher frequency (at $t \approx 700$ s in Fig. 1d, and at $t \approx 560$ s in Fig. 2d). This transition to a higher mean frequency corresponds to a shortening of the mean oscillation period *T*. Thus, the partial synchronisation was achieved by entraining the individual cells to an oscillatory cycle whose period is shorter than that of their free-running oscillations (Fig. 3). In addition, the frequency distribution of the partially synchronized cells was skewed toward lower frequencies; this means that the mean of the distribution lies close to the high frequency end of the distribution and that only very few cells oscillated at a faster pace than the mean (Fig. 3). Taken together, these findings show that the faster oscillating cells entrain the population to their rhythm. This scenario is characteristic of synchronisation by phase advancement.

At intermediate yeast cell densities the cells may have achieved partial synchronisation as reflected by an order parameter *R* that did not come close to unity, and by distributions of frequencies and phases that did not collapse onto unique values. As the immobilized cells were distributed on the bottom of the coverslip in the observation chamber, spatial aspects of the synchronisation need to be considered as well. We observed that during the episode of partial synchronisation, most cells in the field of view showed glycolytic oscillations whose characteristics (frequency, phase) were close to that of the synchronized cells. However, a minority of cells still oscillated at frequencies $f_i$, phases $\phi _i$, or relative phases $\Delta \phi _i$, which may have substantially diverged from those of the almost synchronized population. These were spatially dispersed among the synchronized cells (Fig. 5). This feature is consistent with that reported earlier for intermediate cell densities^13^, and it is more pronounced at decreasing cell densities $\rho$.

A closer inspection of the cells immobilized on the coverslip reveals the formation of spatial clusters, which are characterized by a locally enhanced cell density (or relative cell area density $\sigma _i$). This phenomenon was more pronounced in sparse populations, since, here, the occurrence of almost void areas could be detected (Fig. 6). During partial synchronisation the degree of coherence among the cells belonging to a cluster (as monitored by $R_c$) may exceed that of the entire population, because of a stronger effective coupling among the cells of the cluster. They communicate through the release and uptake of the messenger acetaldehyde^28^, which diffuses through the extracellular medium. In denser clusters, the free distance between adjacent cells is smaller, and so is the decrease in acetaldehyde concentration due to diffusion (and dilution) in the extracellular matrix. Hence, clusters behave as domains where the cell area density is enhanced above that of the entire population.

The enhanced synchrony in the clusters persisted for a few oscillation periods, however, the persistence was always shorter than the episode of partial synchronisation. This indicates that in the clusters the cells were either not packed densely enough or that the number of cells in the cluster was not sufficiently high to ensure the appropriate level of acetaldehyde concentration required for a long-lasting entrainment of the cells. Furthermore, during partial synchronisation, the individual clusters always oscillated at mean phases that differed only moderately from each other, thus providing further support for a barely reached suprathreshold level of extracellular acetaldehyde levels. This also means that the dynamics in the clusters is not independent of that in the other clusters. Therefore, the degree of synchronisation achieved in populations of intermediate cell densities (i.e., $R \approx$ 0.6 and $\approx$ 0.8 for the partially synchronized states in Figs. 1 and 2 for the cell densities of $\rho$ = 0.1% and 0.3%, respectively) did not stem from hugely synchronized clusters oscillating at significantly different mean phases.

During partial synchronisation, we have observed that synchronized glycolytic oscillations may give way to travelling glycolytic waves (Fig. 8). In fact, the occurrence of glycolytic waves in yeast cell colonies was reported very recently^49^. The transition from synchronous glycolytic oscillations to travelling glycolytic waves has previously been observed in yeast cell extracts (of *S. carlsbergensis*) studied in spatially extended reactors^50,51^. The main factor leading to this transition from synchronous oscillations to waves was identified as being the energy charge of the yeast extract^52^. Such a mechanism is most probably also at the origin of the transition from glycolytic oscillations to travelling waves in our yeast cell populations.

During the events of partial synchronisation of glycolytic oscillations, the cells presented a continuous distribution of oscillatory frequencies and phases. In fact, we could not distinguish between two dynamic subpopulations, where one oscillated synchronously whereas the other oscillated at different phases. Thus, we conclude that yeast cell populations performing glycolytic oscillations were unable to form chimera states.

## Conclusions

Partial synchronisation is achieved in yeast cell populations of intermediate cell densities. The route leading to such a partial synchronisation involves a time delay between the synchronisation in the frequencies and that in the phases of the glycolytic oscillations. In fact, the synchronisation of the frequencies of oscillations precedes that of their phases. Furthermore, the cells are entrained to oscillate at slightly higher frequencies, i.e., the cells are entrained by phase advancement. Even in the partially synchronized regime, the glycolytic oscillations fail to collapse on a unique frequency and a unique phase that is common to all cells. This means that in the field of view, most of the cells oscillate synchronously, while a few of them still keep oscillating with their own, different phase and possibly frequency. Although spatial domains of more densely packed cells were found in the batch chamber, these clusters only contributed to transient synchronisation of their member cells; the persistence times of episodes of high synchronisation in a cluster were always significantly shorter than the duration of partial synchronisation of the cell population.

Last but not least, we rule out the formation of chimera states in populations of partially synchronized yeast cells.

## Methods

### Cell preparation, immobilisation, and detection

Cells of the yeast *Saccharomyces carlsbergensis* were cultivated, harvested, washed and stored as described by Weber et al.^13^ Cell suspensions of different densities were prepared, where the density $\rho$ is defined as the dry weight of the cells per volume of suspension in g/100 ml suspension. A suspension of $\rho = 0.1\%$ contains about (10–14) $\times 10^6$ cells $\hbox {ml}^{-1}$.

100 $\mu$l of cell suspension containing different cell densities were prepared and transferred to the batch chamber of 10 mm height and 10 mm internal diameter. Its bottom consisted of a 0.1 mm thick, poly-D-lysine coated coverslip. The chamber was covered by a second (not coated) coverslip, once glycolysis was triggered.

In the batch chamber, the cells were allowed to sediment for at least 20 min, where they were immobilized on the coverslip coated with poly-D-lysine. Then the experiment was started by adding 3 mmol l$^{-1}$ KCN to induce anaerobiosis by blocking the respiration chain in the mitochondria. About 10 min later, 52 mmol l$^{-1}$ glucose were added to trigger glycolysis and induce the metabolic oscillations in the cells. By convention, in the experiments, time $t = 0$ s denotes the onset of the glycolytic oscillations approximately 40–200 s after glucose addition.

The intracellular dynamics were monitored by following the autofluorescence of endogenous reduced nicotinamide adenine dinucleotide (NADH), which serves as an indicator for glycolytic activity^12^. The NADH autofluorescence from single yeast cells was measured in a low-light wide-field fluorescence lifetime imaging microscopy setup, which allows for both a minimum exposure of the yeast cells to light and a pulsating illumination, thus avoiding exposure of the cells to light stress or damage^53,54^. In our studies, the setup is used as a fluorescence microscope, where information stored in the fluorescence intensity is monitored. The batch chamber is mounted in an inverted microscope (Ti Eclipse, Nikon, Düsseldorf, Germany) that is equipped with a 100$\times$ (NA 1.3) S-Fluor objective (Nikon) and a position-sensitive single photon counting photomultiplier tube detector (LINCam 25mm, Photonscore, Magdeburg, Germany). For excitation of intracellular NADH a 8 MHz pulsed frequency-tripled neodymium vanadate laser tuned at 355 nm (HighQ Laser, Hohenems, Austria) was used. The light emitted by the cells was filtered by both a long-pass (LP 442, AHF, Tübingen, Germany) and a bandpass filter (BL 440/40, AHF) and detected by the photomultiplier in the wavelength range of 442 nm $\le \lambda \le$ 465 nm. The positions where the incident photons were collected on the photomultiplier were binned into frames of $512 \times 512$ pixels, resulting in a resolution of 0.33 $\mu$m/pixel in the object space. The field of views had a diameter of 169 $\mu$m, monitoring an area of 2.2$\times 10^4$ $\mu$m$^2$. A time binning of 2 s was chosen as being appropriate to analyse glycolytic oscillations.

### Image analysis

The immobilized yeast cells were randomly distributed on the coverslip. Following the protocols established in our previous work^13,55^, we have analysed the NADH fluorescence signal originating from each of the cells as well as the collective population signal in the field of view. The fluorescence signal from each immobilized cell was monitored in time. At any instant, the fluorescence $F_i$ of the cell *i* is the mean value of the intensity of the fluorescence signal detected in the area occupied by the individual cell, $F_i = a_i^{-1}\sum n_i$, where $n_i$ is the number of incident photons originating from the area $a_i$ occupied by the cell *i*. The temporal sequence $F_i(t)$ yields the time evolution of the fluorescence of an individual cell.

The time-series of the fluorescence of each cell (i.e., $F_i(t)$) was subjected to a baseline subtraction to eliminate any spurious drifts or long-term trends. The baseline was computed as the walking average of the fluorescence data using a time window of $\delta t$ = 200 s corresponding to about 3 to 5 periods of oscillation (of typically from 40 s to 60 s per period). The resulting baseline-subtracted time-series of the fluorescence of a single cell were centred around 0, i.e., the fluorescence oscillated around 0. Noise reduction was achieved by bandpass filtering. The applied Fourier bandpass filter cut off frequencies higher than 40.0 mHz and lower than 14.0 mHz. Thus, the frequencies of the glycolytic oscillations were preserved in the filtered time series $x_i(t)$^13,55^.

The collective signal (collective fluorescence) of the entire cell population *X*(*t*) was obtained by averaging over all baseline subtracted time series $x_i(t)$ of the individual cells.

The amplitudes of the oscillations were measured using the baseline-subtracted fluorescence time-series. For a more convenient scaling, we present the amplitude data as normalized amplitudes $\tilde{A_i}$, which were obtained by dividing the amplitudes $x_i(t)$ of the oscillations by the oscillation of maximum amplitude $x_{i,max} = maximum(|x_i|)$ measured for each cell, such that $\tilde{A_i} = x_i / x_{i,max}$ is in the range $[-1, 1]$.

### Analysis of synchronicity within a cell population

The phase $\phi _i(t)$ of each oscillating cell *i*

1 $$\begin{aligned} \phi _i(t)=\arctan \left( \frac{{\widetilde{x}}_i(t)}{x_i(t)}\right) \end{aligned}$$

was calculated from the filtered time-series of the fluorescence signal $x_i(t)$ and ${\widetilde{x}}_i(t)$ that was computed through the Hilbert transform

2 $$\begin{aligned} {\widetilde{x}}_i(t)=\frac{1}{\pi }PV\int ^{\infty }_{-\infty } \frac{x_i(t')}{t-t'} dt' \end{aligned}$$

using the Matlab (MathWorks Inc., Natick, MA, USA) functions ’angle’ and ’hilbert’. In Eq. (2) PV denotes that the integral should be evaluated as the Cauchy principle value.

The instantaneous frequencies $f_i(t)$ of each cell were computed as the time derivatives of the unwrapped phases. The latter were smoothed by applying a sliding window of width $\delta t$ = 40 s.

The order parameter *R*^56^

3 $$\begin{aligned} R(t)=\left| \frac{1}{N} \sum ^N_i{e^{i\phi _i(t)}} \right| \end{aligned}$$

was chosen for measuring the phase synchronisation among the cells at each time point. An order parameter $R = 1$ indicates a complete synchronisation among the cells in a population, whereas $R = 0$ means that the cells oscillate at random phases.

The macroscopic oscillation phase $\Phi$ was obtained as

4 $$\begin{aligned} \Phi = \arg \left( \frac{1}{N} \sum ^N_i{e^{i\phi _i(t)}} \right) . \end{aligned}$$

To characterize the behaviour of individual cells with respect to the collective dynamics of the population, we determined the instantaneous phase shift $\Delta \phi _i$ of the phase of cell *i*

5 $$\begin{aligned} \Delta \phi _i(t) = \phi _i(t) - \Phi (t) \end{aligned}$$

with respect to the phase of the global macroscopic oscillations $\Phi$. $\Delta \phi _i$ was computed using the unwrapped versions of the phases $\phi _i$ and $\Phi$.

The relative order parameter $R_i(t)$ of each individual cell with respect to the global order parameter *R*(*t*) was calculated as

6 $$\begin{aligned} R_i(t)=\left| \frac{1}{2} \left( {e^{i\phi _i(t)}} + {e^{i \Phi (t)}} \right) \right| . \end{aligned}$$

Again, the values of the relative order parameter $R_i$ range from 0 to 1, in analogy to those of the order parameter *R* (Eq. 3). The determination of $R_i$ allows for visualizing spatial aspects of the coherence among the oscillating cells in the population. Together, the instantaneous phase shift $\Delta \phi _i$ and the relative order parameter $R_i$ indicate how different or similar a cell behaved compared to the dynamics of the global population.

The distributions of the frequencies of the oscillations were obtained by binning the frequencies in intervals of $\delta f$ = 0.50 mHz. The binning of the distribution of the phases $\phi _i$ and relative phases $\Delta \phi _i$ was calculated using interrogation windows of width $\delta \phi$ = 0.05 $\pi$. These distributions were determined for each instant in time.

### Detection of clusters of cells

As described in “Cell preparation, immobilisation, and detection” section, cells were allowed to sediment on the poly-D-lysine-coated coverslip, where they were immobilized, leading to a random positioning of the cells on the coverslip. This procedure may lead to the formation of clusters where cells are locally more densely packed on some domains of the coverslip. To detect any clusters, we performed a cluster search based on a Voronoi tessellation to determine the area $S_i$ occupied by each cell using the Matlab routine ’polyarea’. The Voronoi polygons (determined using the Matlab routines ’voronoi’ and ’delaunayTriangulation’) encompass all points that are closer to the centre of the cell *i* under consideration than to any other cell. The cell area density $\tilde{\sigma _i}$ is obtained as the reciprocal of the area $S_i$ occupied by cell *i*, i.e., $\tilde{\sigma _i}$ = 1/$S_i$. For further analyses, we use the relative cell area density $\sigma _i$ , which is given as

7 $$\begin{aligned} \sigma _i = \frac{\tilde{\sigma _i}}{\frac{1}{N} \sum ^N_i{\tilde{\sigma _i}}} , \end{aligned}$$

i.e., the cell area density expressed in units of the mean cell area density^57,58^.

Spatial clusters of cells were detected by preselecting the cells, either by detecting the narrow spatial domains of closely packed cells, i.e., cells that possess a large relative cell area density $\sigma _i$ (corresponding to low $S_i$), or as closely packed domains of cells *i* whose distance $r_{ij}$ = $\sqrt{(x_i - x_j)^2 + (y_i - y_j)^2}$ (where $x_i,y_i$ and $x_j,y_j$ are the coordinates of the centres of cells *i* and *j*) to its neighbours *j* did not exceed a certain threshold. We chose cells whose area density $\sigma _i > median(\sigma _i)$ and whose distances $r_{ij} \le$ 6 $\mu$m as belonging to a spatial cluster. These cells were segmented into clusters using the Matlab routine ’clusterdata’. The number of clusters was preselected as ($\approx N/10$), where *N* is the number of cells in the field of view.

To assess whether and to which extent cells that belong to a spatial cluster also synchronized their dynamics which each other during the events of partial synchronisation, we introduce the cluster order parameter $R_c$

8 $$\begin{aligned} R_c(t)=\left| \frac{1}{M} \sum ^M_i{e^{i\phi _i(t)}} \right| \end{aligned}$$

in analogy to the order parameter *R* (Eq. 3). The cluster order parameter $R_c$ is a measure for the degree of synchronisation among the *M* cells that belong to a spatial cluster. In our analyses, we considered that cells of a cluster are synchronized, once $R_c$ exceeded a threshold that was at least 0.1 higher than the order parameter (i.e., $R_c \ge R$ + 0.1). For such events of synchronisation among the cells of a cluster, we have mapped the phases $\phi _i$ of the cells onto their positions, in order to evaluate any spatial aspects of the synchronisation.

### Test for chimera states

In order to test whether chimera states were formed during partial synchronisation of yeast cells, we monitored the development of the distributions of the relative phase $\Delta \phi _i$ in time. At an instant where the cells displayed partial synchronisation, the distributions were skewed Gaussians. We then cut off the tails of the distribution, and subsequently exclusively monitored the dynamics of those cells whose relative phases laid within the narrow interval $- \frac{1}{12} \pi \le \Delta \phi _i \le \frac{1}{12} \pi$, and neglected all others. This means that we focused on the dynamics of the cells whose phases diverged only slightly from the common phase of the synchronized signal. In a chimera, the phases of these cells should remain closely together, since they represent the ordered, synchronized part of the chimera.

## Supplementary information

Supplementary information.

Supplementary Video S1.

Supplementary Video S2.

Supplementary Video S4.

Supplementary Video S5.

## Data Availability

The datasets generated and analysed during the current study are available from the corresponding author on reasonable request.

## References

[1] Winfree AT (2001). The Geometry of Biological Time.

[2] Kim J-R, Shin D, Jung SH, Heslop-Harrison P, Cho K-H (2010). A design principle underlying the synchronization of oscillations in cellular systems. J. Cell Sci..

[3] Goldbeter A (2017). Dissipative structures and biological rhythms. Chaos.

[4] Goldbeter A (1996). Biochemical Oscillations and Cellular Rhythms: The Molecular Bases of Periodic and Chaotic Behaviour.

[5] Maroto M, Monk NAM (2008). Cellular Oscillatory Mechanisms.

[6] Rapp P (1979). An atlas of cellular oscillators. J. Exp. Biol..

[7] Schaefer U, Boos W, Takors R, Weuster-Botz D (1999). Automated sampling device for monitoring intracellular metabolite dynamics. Anal. Biochem..

[8] Duysens LN, Amesz J (1957). Fluorescence spectrophotometry of reduced phosphopyridine nucleotide in intact cells in the near-ultraviolet and visible region. Biochim. Biophys. Acta.

[9] Betz A, Chance B (1965). Phase relationship of glycolytic intermediates in yeast cells with oscillatory metabolic control. Arch. Biochem. Biophys..

[10] Ghosh A, Chance B (1964). Oscillations of glycolytic intermediates in yeast cells. Biochem. Biophys. Res. Commun..

[11] Ghosh A, Chance B, Pye E (1971). Metabolic coupling and synchronization of nadh oscillations in yeast cell populations. Arch. Biochem. Biophys..

[12] Hess B, Boiteux A (1968). Mechanism of glycolytic oscillation in yeast. i. aerobic and anaerobic growth conditions for obtaining glycolytic oscillation. Hoppe Seylers Z. Physiol. Chem..

[13] Weber A, Prokazov Y, Zuschratter W, Hauser MJB (2012). Desynchronisation of glycolytic oscillations in yeast cell populations. PLoS ONE.

[14] Aon MA, Cortassa S, Westerhoff HV, van Dam K (1992). Synchrony and mutual stimulation of yeast cells during fast glycolytic oscillations. J. Gen. Microbiol..

[15] Richard P, Bakker BM, Teusink B, Van Dam K, Westerhoff HV (1996). Acetaldehyde mediates the synchronization of sustained glycolytic oscillations in populations of yeast cells. Eur. J. Biochem..

[16] Richard P (2003). The rhythm of yeast. FEMS Microbiol. Rev..

[17] Danø S, Sørensen PG, Hynne F (1999). Sustained oscillations in living cells. Nature.

[18] Poulsen AK, Petersen MØ, Olsen LF (2007). Single cell studies and simulation of cell-cell interactions using oscillatory glycolysis in yaest cells. Biophys. Chem..

[19] Özalp VC, Pedersen TR, Nielsen LJ, Olsen LF (2010). Time-resolved measurements of intracellular atp in the yeast saccharomyces cerevisiae using a new type of nanobiosensor. J. Biol. Chem..

[20] Gustavsson A-K (2012). Sustained glycolytic oscillations in individual isolated yeast cells. FEBS J..

[21] Thoke HS (2015). Tight coupling of metabolic oscillations and intracellular water dynamics in saccharomyces cerevisiae. PLoS ONE.

[22] Lipkin EW, Teller DC, de Haën C (1983). Dynamic aspects of insulin action: synchronization of oscillatory glycolysis in isolated perfused rat fat cells by insulin and hydrogen peroxide. Biochemistry.

[23] Chou H, Berman N, Ipp E (1992). Oscillations of lactate released from islets of langerhans: evidence for oscillatory glycolysis in beta-cells. Am. J. Physiol. Endocrinol. Metabol..

[24] Yang J-H, Yang L, Qu Z, Weiss JN (2008). Glycolytic oscillations in isolated rabbit ventricular myocytes. J. Biol. Chem..

[25] Amemiya T (2017). Primordial oscillations in life: direct observation of glycolytic oscillations in individual hela cervical cancer cells. Chaos.

[26] Chandra FA, Buzi G, Doyle JC (2011). Glycolytic oscillations and limits on robust efficiency. Science.

[27] Palková Z, Váchová L (2006). Life within a community: benefit to yeast long-term survival. FEMS Microbiol. Rev..

[28] Lushchak OV, Müller SC, Mair T (2006). Comparison of glycolytic nadh oscillations in yeasts saccharomyces cerevisiae and saccharomyces carlsbergensis. Ukr. Biochem. J..

[29] Schütze J, Mair T, Hauser MJB, Falcke M, Wolf J (2011). Metabolic synchronization by traveling waves in yeast cell layers. Biophys. J..

[30] Bolyó J, Mair T, Kuncová G, Hauser MJB (2010). Spatiotemporal dynamics of glycolytic waves provides new insights into the interactions between immobilized yeast cells and gels. Biophys. Chem..

[31] Amemiya T (2015). Collective and individual glycolytic oscillations in yeast cells encapsulated in alginate microparticles. Chaos.

[32] Aldridge J, Pye EK (1976). Cell density dependence of oscillatory metabolism. Nature.

[33] Pikovsky A, Rosenblum M, Kurths J (2004). Synchronization: A Universal Concept in Nonlinear Science.

[34] Strogatz SH (2000). From kuramoto to crawford: exploring the onset of synchronization in populations of coupled oscillators. Physica D.

[35] Schwab DJ, Baetica A, Mehta P (2012). Dynamical quorum-sensing in oscillators coupled through an external medium. Physica D.

[36] De Monte, S., d’Ovidio, F., Danø, S. & Sørensen, P. G. Dynamical quorum sensing: population density encoded in cellular dynamics. *Proc. Natl. Acad. Sci. U.S.A.* **104**, 18377–18381. 10.1073/pnas.0706089104 (2007). 10.1073/pnas.0706089104 PMC2141785 18003917

[37] Taylor AF, Tinsley MR, Wang F, Huang Z, Showalter K (2009). Dynamical quorum sensing and synchronization in large populations of chemical oscillators. Science.

[38] Wang S-W, Tang L-H (2019). Emergence of collective oscillations in adaptive cells. Nat. Commun..

[39] Gustavsson A-K, Adiels CB, Mehlig B, Goksör M (2015). Entrainment of heterogeneous glycolytic oscillations in single cells. Sci. Rep..

[40] English LQ (2008). Synchronization of oscillators: an ideal introduction to phase transitions. Eur. J. Phys..

[41] Peter F, Pikovsky A (2018). Transition to collective oscillations in finite kuramoto ensembles. Phys. Rev. E.

[42] Kuramoto Y, Battogtokh D (2002). Coexistence of coherence and incoherence in nonlocally coupled phase oscillators. Nonlinear Phenom. Complex Syst..

[43] Abrams DM, Strogatz SH (2004). Chimera states for coupled oscillators. Phys. Rev. Lett..

[44] Panaggio MJ, Abrams DM (2015). Chimera states: coexistence of coherence and incoherence in networks of coupled oscillators. Nonlinearity.

[45] Tinsley MR, Nkomo S, Showalter K (2012). Chimera and phase-cluster states in populations of coupled chemical oscillators. Nat. Phys..

[46] Martens EA, Thutupalli S, Fourrière A, Hallatschek O (2013). Chimera states in mechanical oscillator networks. Proc. Natl. Acad. Sci. U.S.A..

[47] Wickramasinghe M, Kiss IZ (2014). Spatially organized partial synchronization through the chimera mechanism in a network of electrochemical reactions. Phys. Chem. Chem. Phys..

[48] Totz JF, Rode J, Tinsley MR, Showalter K, Engel H (2018). Spiral wave chimera states in large populations of coupled chemical oscillators. Nat. Phys..

[49] Mojica-Benavides, M. *et al.* Intercellular communication induces glycolytic synchronization waves between individually oscillating cells. arXiv:1909.05187v2 (2020). 10.1073/pnas.2010075118 PMC8017953 33526662

[50] Mair T, Müller SC (1996). Travelling nadh and proton waves during oscillatory glycolysis in vitro. J. Biol. Chem..

[51] Bagyan S, Mair T, Suchorski Y, Hauser MJB, Straube R (2008). Spatial desynchronization of glycolytic waves as revealed by karhunen-loève analysis. J. Phys. Chem. B.

[52] Zymányi L, Khoroshyy P, Mair T (2010). A chemometric method to identify enzymatic reactions leading to the transition from glycolytic oscillation to waves. Physica D.

[53] Guerin, B. & Jacques, R. Photoinhibition de l’adaptation respiratoire chez saccharomyces cerevisiae ii. le spectre d’action. *Biochim. Biophys. Acta* **153**, 138–142. 10.1016/0005-2728(68)90154-0 (1968). 10.1016/0005-2728(68)90154-0 5638382

[54] Bodvard K (1813). Continuous light exposure causes cumulative stress that affects the localization oscillation dynamics of the transcription factor msn2p. Biochim. Biophys. Acta.

[55] Weber A, Prokazov Y, Zuschratter W, Hauser MJB, Müller SC, Plath PJ, Radons G, Fuchs A (2018). From synchronised to desynchronised glycolytic oscillations in individual yeast cells. Complexity and Synergetics.

[56] Shinomoto S, Kuramoto Y (1986). Phase transitions in active rotator systems. Prog. Theor. Phys..

[57] Ebeling H, Wiedemann G (1993). Detecting structure in two dimensions combining vorornoi tessellation and percolation. Phys. Rev. E.

[58] Ramella M, Boschin W, Fadda D, Nonino M (2001). Finding galaxy clusters using voronoi tessellations. Astron. Astrophys..

