# Supplementary material for: Partial synchronisation of glycolytic oscillations in yeast cell populations
Source: Sci Rep. 2020 Nov 12;10:19714. doi: 10.1038/s41598-020-76242-8 (PMC7661732; doi:10.1038/s41598-020-76242-8)
Supplement: Supplementary file 1 — Supplementary information. [file 41598_2020_76242_MOESM1_ESM.pdf]

Partial synchronisation of glycolytic oscillations in yeast cell populations
Supplementary Information

André Weber [1], Werner Zuschratter [1], and Marcus J. B. Hauser [2]

[1] *Combinatorial NeuroImage Core Facility,*
Leibniz Institute for Neurobiology Magdeburg,
Brennekestraße 6, 39120 Magdeburg, Germany

[2] *Institute of Biology, Otto-von-Guericke Universität Magdeburg,*
Pfälzer Straße 5, 39106 Magdeburg, Germany

(Dated: November 3, 2020)

For further illustration, we provide supplementary material on the partial synchronisation of glycolytic oscillations in yeast cells. Four videos show the dynamics of yeast cell populations at cell densities of $\rho = 0.1\%$ and 0.3%, respectively. The videos stem from the experiments shown in Figs. 1 and 2 (of the paper), respectively. While for the cell population at $\rho = 0.1\%$ the field of view has a diameter of 169 μm, the field of view for the cell population at $\rho = 0.3\%$ was chosen as 85 μm $\times$ 85 μm. Two of the videos focus on the individual dynamics of the cells in the population (Fig. S1 and Video S1, as well as Fig. S4 and Video S4) and two videos show the dynamics in local clusters (Fig. S2 and Video S2 as well as Fig. S5 and Video S5). We provide further documentation that the oscillation amplitudes of glycolytic oscillations in the individual cells already started to decay before the partial synchronisation among the cells was achieved (Figs. S3 and S6).

For completeness, we also include data for the denser cell population (at $\rho = 0.3\%$) in analogy to Figs. 4 and 5 from the paper. Fig. S7 shows the phases of the oscillations of each cell on the unit circle for both the asynchronous and the partially synchronized cells. A plot of the distribution of phases and of the relative order parameter R_i from both the asynchronous and the partially synchronized cells is shown in Fig. S8. These figures are analogous to Figs. 4 and 5 shown in the paper. Furthermore, the dependence of the oscillations on the spatial position of the cells is investigated. Therefore, local clusters of densely packed cells are segmented according to their distance r_{ij} (Fig. S9b) and relative cell area density σ_i (Fig. S9c), analogously to Fig 6 shown in the paper. The time dependence of the local order parameter R_c of each cluster is plotted (Fig. S10b,d) in analogy to Fig. 7 shown in the paper.

Finally, we include data on the behaviour of yeast populations whose cell density lies outside the range that leads to partial synchronisation (i.e., $0.08\% \leq \rho \leq 0.3\%$). At high cell densities (e.g., at $\rho = 0.7$ %) the cells achieve a complete synchronisation of their glycolytic oscillations to a common rhythm (Fig. S11), while for low cell densities (e.g., $\rho = 0.001\%$) not even a partial synchronisation is observed (Fig. S12).

I. DYNAMICS OF A SPARSE CELL POPULATION OF DENSITY $\rho = 0.1\%$

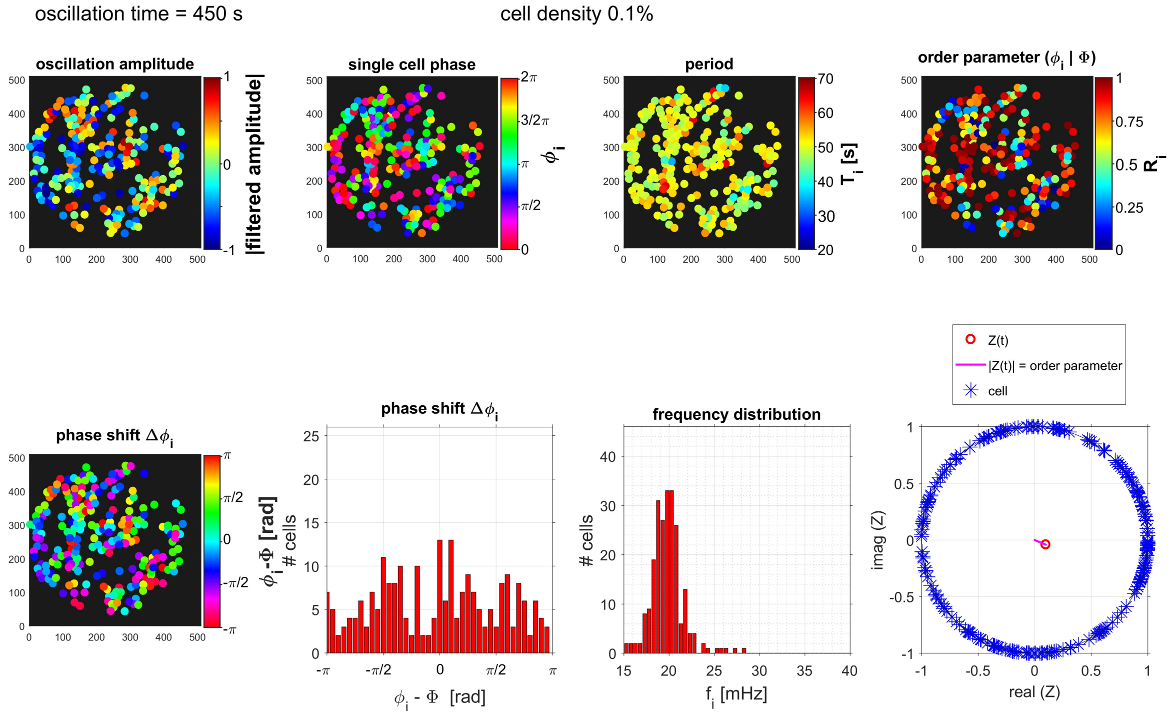

FIG. S1. (**Video S1**) Analysis of the dynamics of a yeast population of cell density $\rho = 0.1\%$. The field of view had a diameter of 169 μm hosting 232 cells. *Top row (from left to right)*: (i) Amplitudes of the oscillations of the individual cells in the field of view, (ii) their phases ϕ_i, (iii) the periods T_i of oscillations, and (iv) the relative order parameter R_i of the individual cells. *Bottom row (from left to right)*: (v) Phase shift $\Delta\phi_i$ of the individual cells with respect to the phase of the collective dynamics, (vi) histogram of $\Delta\phi_i$, (vii) distribution of frequencies f_i of the cells, and (viii) the distribution of phases ϕ_i of the glycolytic oscillations plotted on the unit circle; the phase position of each cell is marked by an asterisk. The vector starting at the origin points to the value of the average phase Φ, and the magnitude of the vector corresponds to the value of the order parameter R.

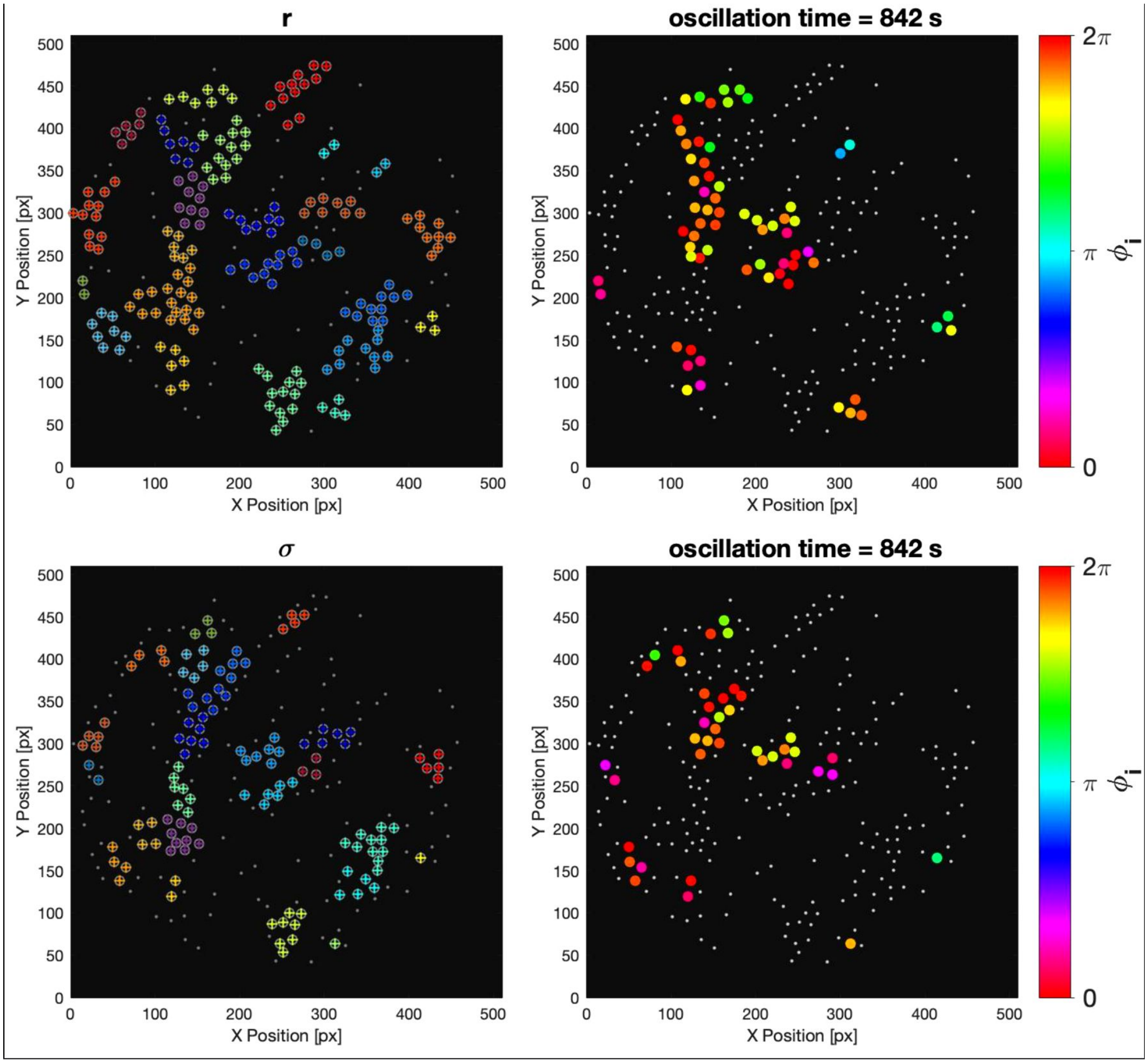

FIG. S2. (**Video S2**) Temporal evolution of oscillation phases of cells in a cluster with enhanced synchronisation for the cell population of density $\rho = 0.1\%$. *Left column:* Positions of all cells (dots) and cells belong to clusters (crosses) obtained due to segmentation with respect to (*top row*) the (shortest) intracellular distances $r_{ij} \leq 6\ \mu$m and (*bottom row*) to the (highest) relative cell area density $\sigma_i > median(\sigma) = 2.53$ (shown in Fig. 6 in the paper). *Right column (from top to bottom):* Maps of clusters with enhanced synchrony during partial synchronisation. When the cluster order parameter exceeded the threshold of $R_c \geq 0.75$, the phases ϕ_i of the cells were mapped onto their positions, else the cells are indicated by dots. Clusters obtained by segmentation with respect (*top row*) to r_{ij} and (*bottom row*) to σ_i.

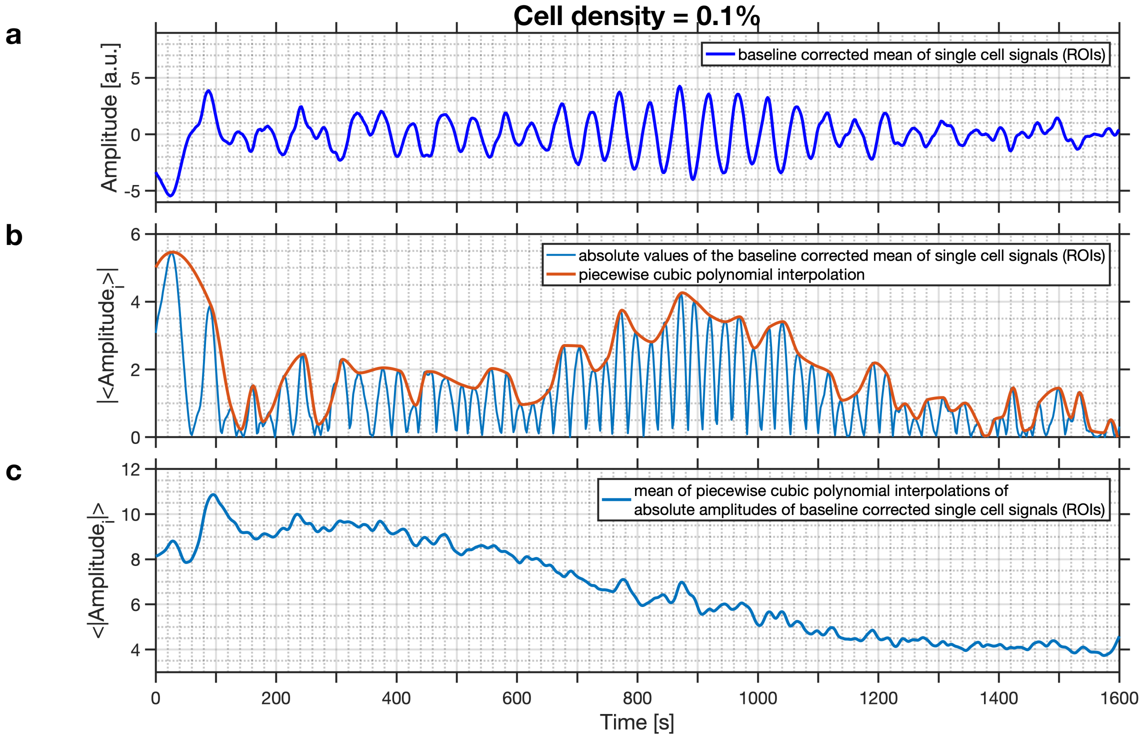

FIG. S3. (a) Time series of the collective NADH fluorescence signal for a yeast cell population of density $\rho = 0.1\%$ (see also Fig. 1a). (b) Absolute value of the global glycolytic oscillations (blue trace). The maxima of the absolute values of these oscillations were detected using the Matlab routine 'findpeaks' and their envelope (orange line) was obtained using a shape-preserving piecewise cubic interpolation implemented in the Matlab routine 'pchip'. (c) Averaged value of the interpolated absolute amplitudes of the glycolytic oscillations of the individual cells. These were obtained using the same protocol as for the collective fluorescence signal. It is observed that the absolute amplitude of the oscillations of the individual cells begins to decay after $t \approx 400$ s, in good agreement with the oscillation amplitudes presented in the heat map of Fig. 1b.

II. DYNAMICS OF A DENSER CELL POPULATION OF DENSITY $\rho = 0.3\%$

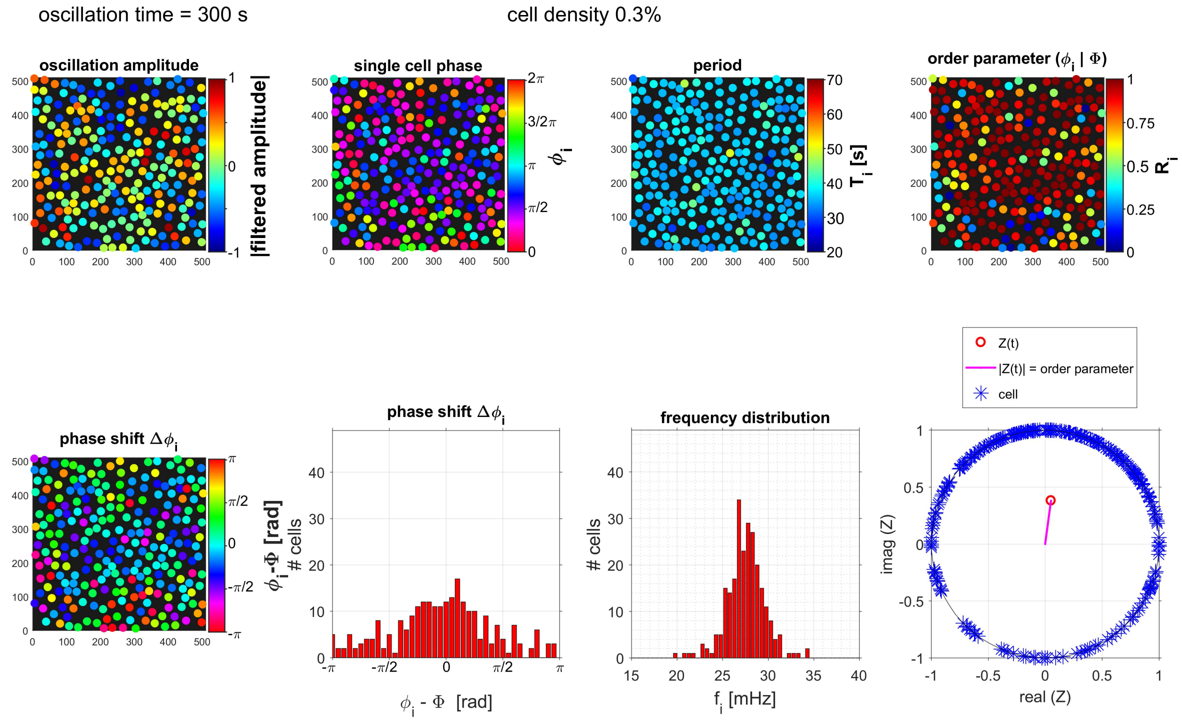

FIG. S4. (**Video S4**) Analysis of the dynamics of a yeast population of cell density $\rho = 0.3\%$. The field of view had a size of $85 \times 85\,\mu\text{m}^2$ contained 251 cells. *Top row (from left to right)*: (i) Amplitudes of the oscillations of the individual cells in the field of view, (ii) their phases ϕ_i, (iii) the periods T_i of oscillations, and (iv) the relative order parameter R_i of the individual cells. *Bottom row (from left to right)*: (v) Phase shift $\Delta\phi_i$ of the individual cells with respect to the phase of the collective dynamics, (vi) histogram of $\Delta\phi_i$, (vii) distribution of frequencies f_i of the cells, and (viii) the distribution of phases ϕ_i of the glycolytic oscillations plotted on the unit circle; the phase position of each cell is marked by an asterisk. The vector starting at the origin points to the value of the average phase Φ, and the magnitude of the vector corresponds to the value of the order parameter R.

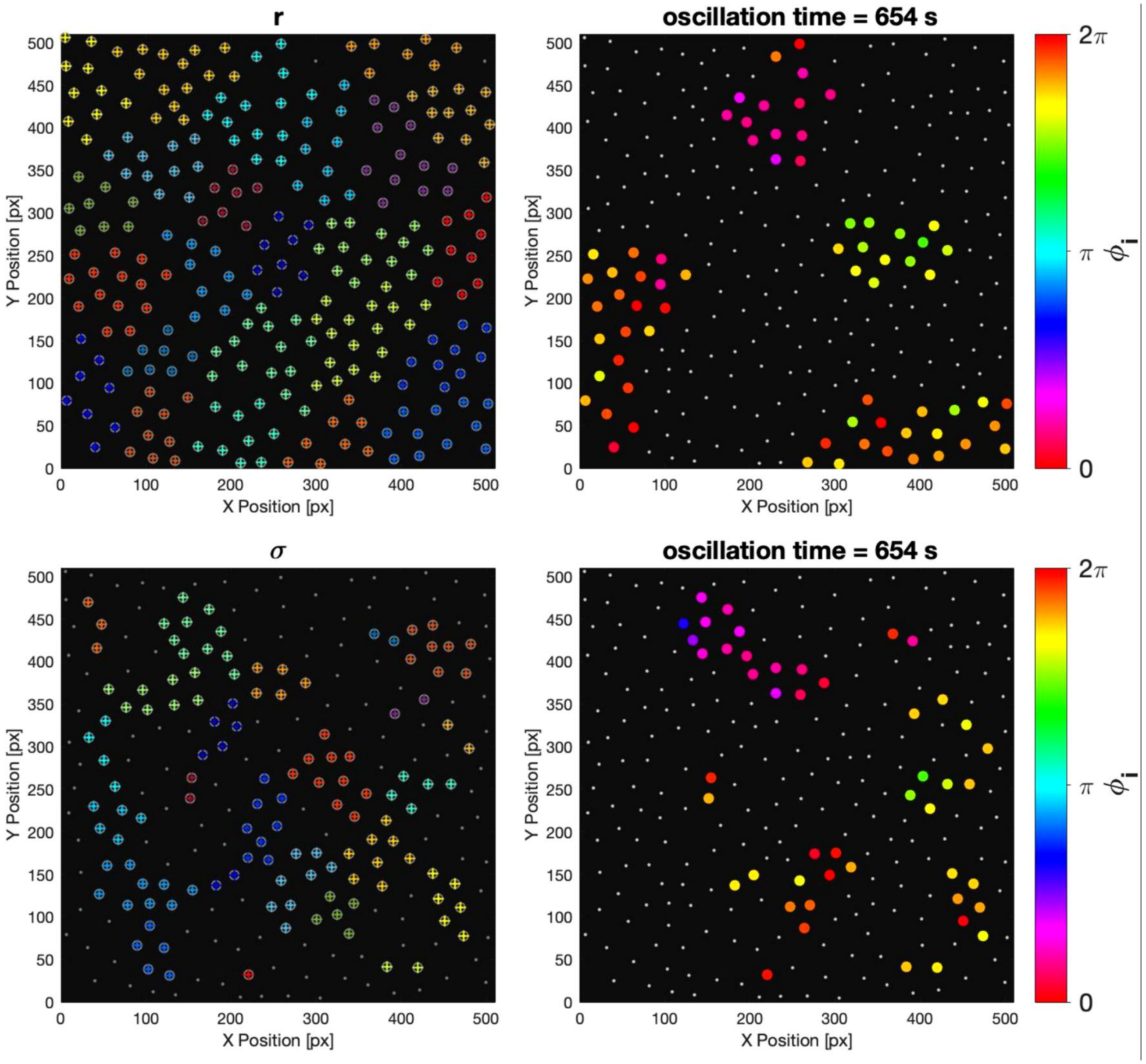

FIG. S5. (**Video S5**) Temporal evolution of oscillation phases of cells in a cluster with enhanced synchronisation for the cell population of density $\rho = 0.3\%$. *Left column:* Positions of all cells (dots) and cells belong to clusters (crosses) obtained due to segmentation with respect to (*top row*) the (shortest) intercellular distances $r_{ij} \leq 6\mu m$ and (*bottom row*) to the (highest) relative cell area density $\sigma_i > median(\sigma) = 12.28$ (shown in Fig. S9). *Right column:* Maps of clusters with enhanced synchrony during partial synchronisation. When the cluster order parameter exceeds the threshold of $R_c \geq 0.9$, the phases ϕ_i of the cells were mapped onto their positions, else the cells are indicated by dots. Clusters obtained by segmentation with respect (*top row*) to r_{ij} and (*bottom row*) to σ_i.

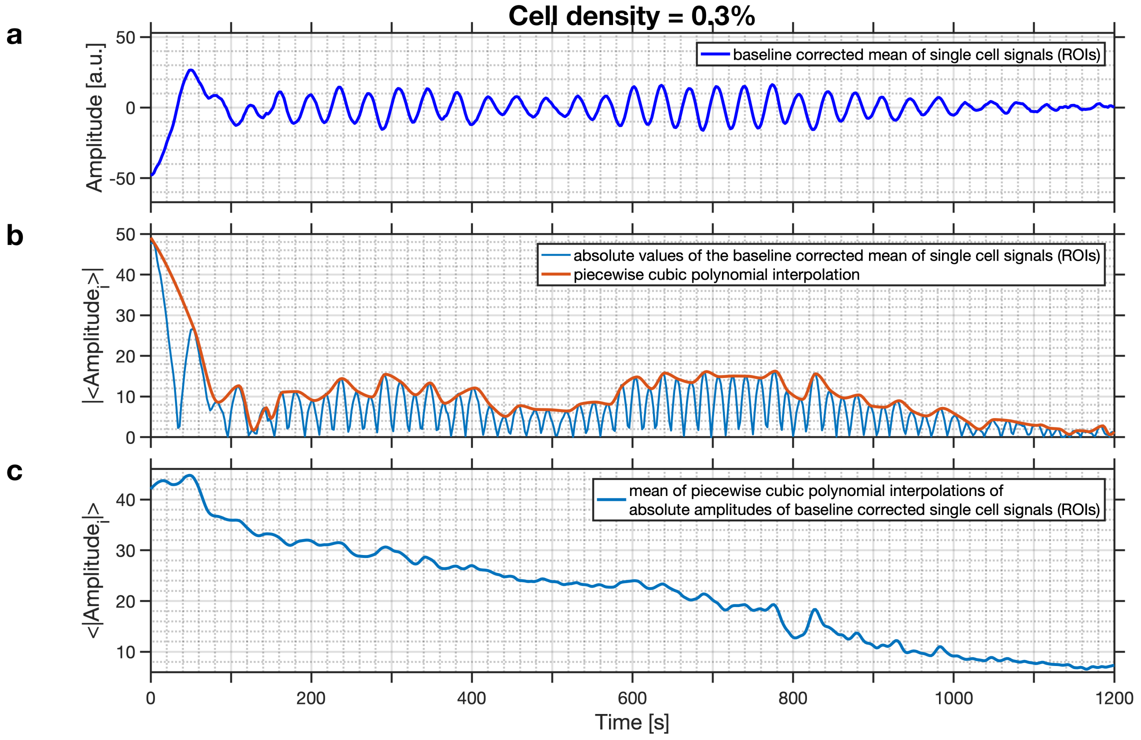

FIG. S6. (a) Time series of the collective NADH fluorescence signal for a yeast cell population of density $\rho =$ 0.3% (see also Fig. 2a). (b) Absolute value of the global glycolytic oscillations (blue trace). The maxima of the absolute values of these oscillations were detected using the Matlab routine 'findpeaks' and their envelope (orange line) was obtained using a shape-preserving piecewise cubic interpolation implemented in the Matlab routine 'pchip'. (c) Averaged vaule of the interpolated absolute amplitudes of the glycolytic oscillations of the individual cells. These were obtained using the same protocol as for the collective fluorescence signal. It is observed that the absolute amplitude of the oscillations of the individual cells decays from the very start of the experiment, in good agreement with the oscillation amplitudes presented in the heat map of Fig. 2b.

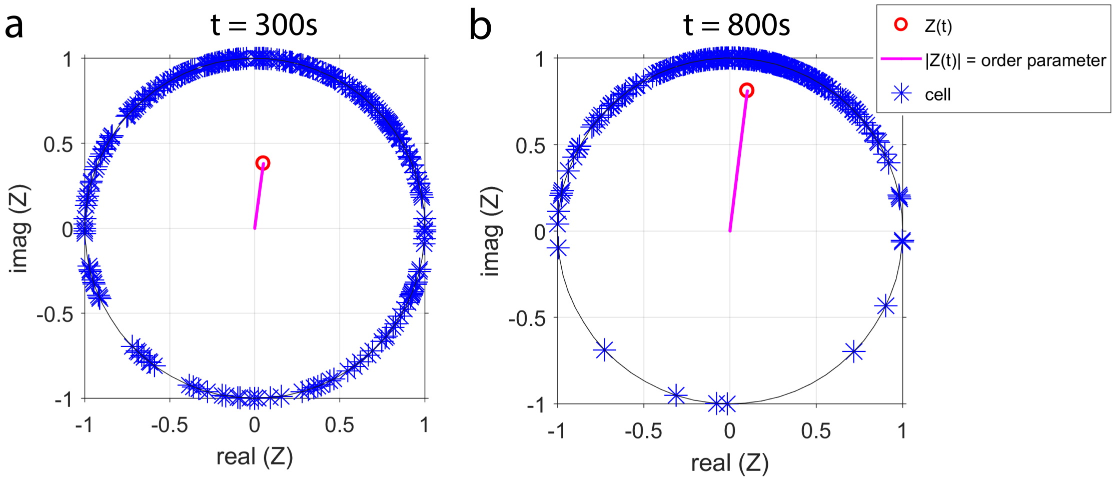

FIG. S7. The phases ϕ_i of the glycolytic oscillations of every cell of a population of cell density $\rho = 0.3\%$ (Fig. 2 in the paper) are plotted on the unit circle (a) during the regime of asynchronous oscillations at $t =$ 300 s and (b) during partial synchronisation at $t = 800$ s. The position of the phase of each cell is marked by an asterisk. The vector starting at the origin points to the value of the average phase Φ, and the magnitude of the vector corresponds to the value of the order parameter R (which is (a) $R = 0.38$ and (b) $R = 0.80$, respectively).

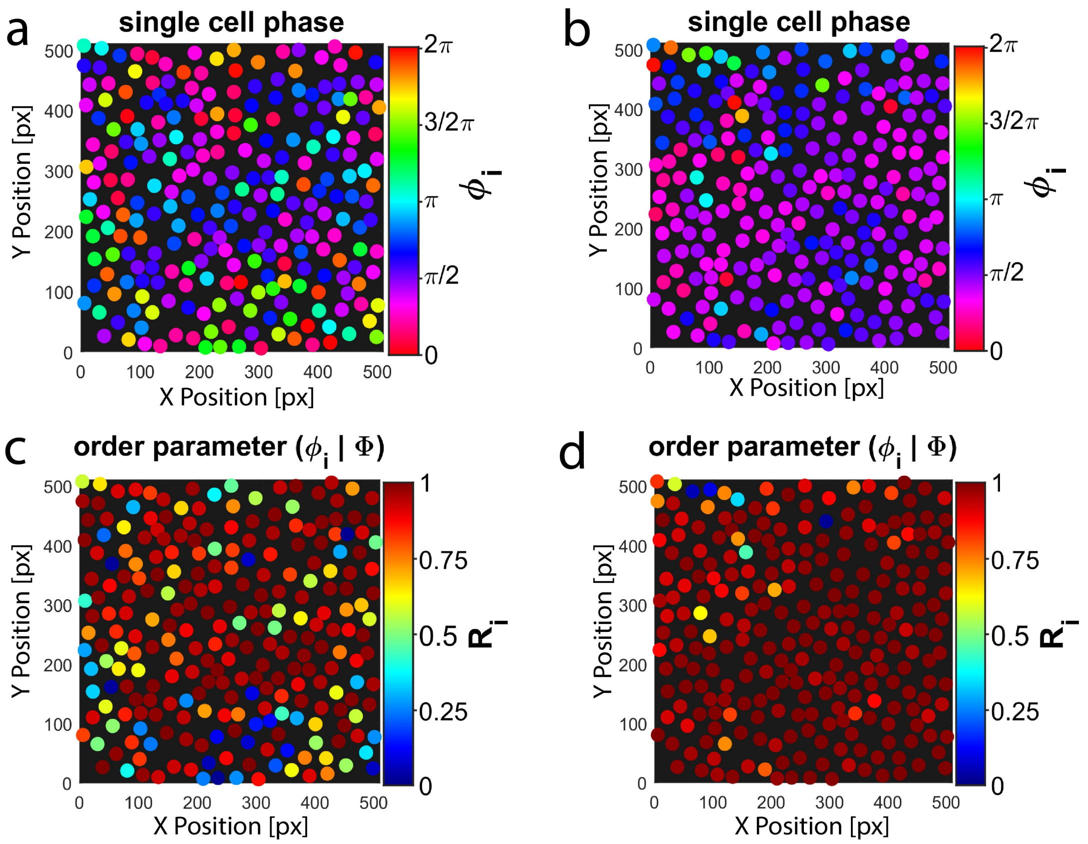

FIG. S8. Spatial partition of the phases ϕ_i of the glycolytic oscillations of each cell in a dense cell population ($\rho = 0.3\%$) (a) during the regime of asynchronous oscillations ($t = 300$ s) and (b) during the partially synchronized state (at $t = 800$ s). Spatial partition of the relative order parameter R_i (c) during the episode of asynchronous oscillations ($t = 300$ s) and (d) during the partially synchronized state (at $t = 800$ s). Data from the experiment at cell density $\rho = 0.3\%$ is shown in Fig. 2 (in the paper). The field of view had a size of $85 \times 85\,\mu\text{m}^2$and it was binned into 512 px $\times$ 512 px, yielding a resolution of 0.167 μm px^{-1}. The field of view contained 251 cells.

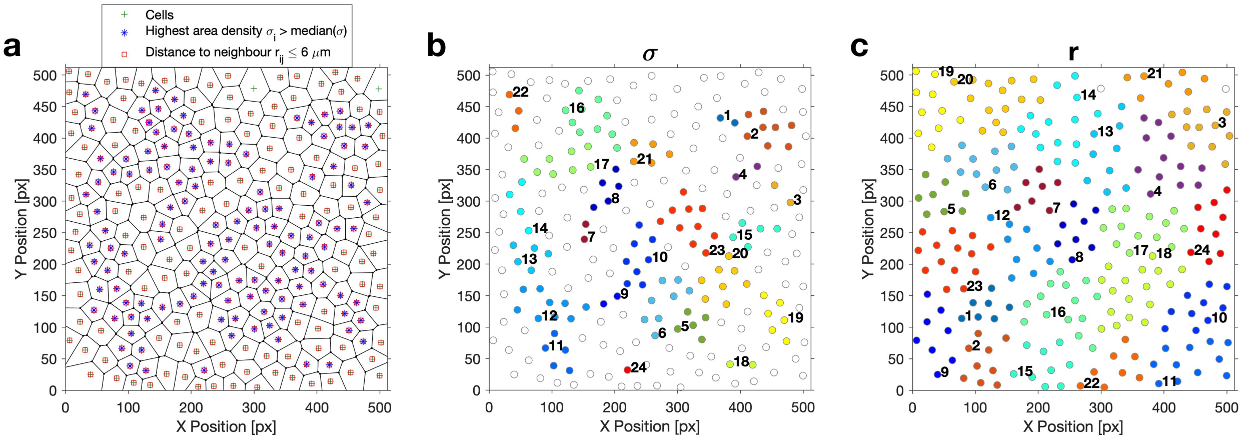

FIG. S9. Voronoi tessellation and segmentation of the cells into spatial clusters. Cell density $\rho = 0.3\%$. (a) In the Voronoi diagram, the positions of the centres of mass of the cells are indicated by green crosses; the cells displaying the highest relative cell area densities $\sigma_i > median(\sigma) = 12.28$ and those with the shortest intercellular distances $r_{ij} \leq 6\mu$m are indicated by blue asterisks and red squares, respectively. (b) Clusters obtained due to segmentation with respect to the (highest) relative cell area density σ_i and (c) with respect to the (shortest) intercellular distances r_{ij}. The field of view had a size of 85 μm $\times$ 85 μm and it was binned into 512×512 pixels. Each pixel had a resolution of 0.167 μm $\times$ 0.167 μm.

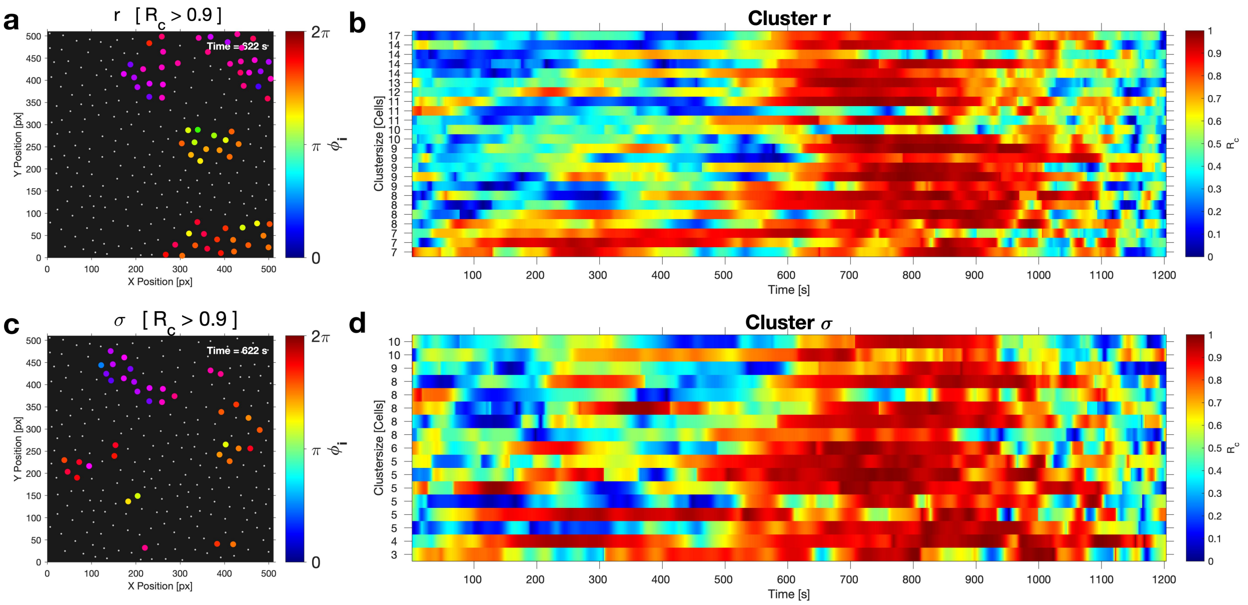

FIG. S10. Maps of cells in a cluster with enhanced synchronisation and cluster order parameter R_c for each of the clusters in the cell population of density $\rho = 0.3\%$ (shown in Fig. S9). (a,c) Maps of clusters with enhanced synchrony during partial synchronisation. When the cluster order parameter exceed the threshold of $R_c \geq 0.9$, the phases ϕ_i of the cells were mapped onto their positions, else the cells are indicated by a point. Clusters are obtained by segmentation with respect (a) to r_{ij} and (c) to σ_i. Video S5 compiles the time evolution of the dynamics in the spatial clusters. (b,d) Time dependence of the order parameter R_c of cell clusters sorted by cluster size. R_c is colour-coded and each line represents R_c of a single cluster over time. The clusters are segmented with respect to (b) r_{ij} and (d) to σ_i.

III. DYNAMICS OF SPARSE AND DENSE CELL POPULATIONS

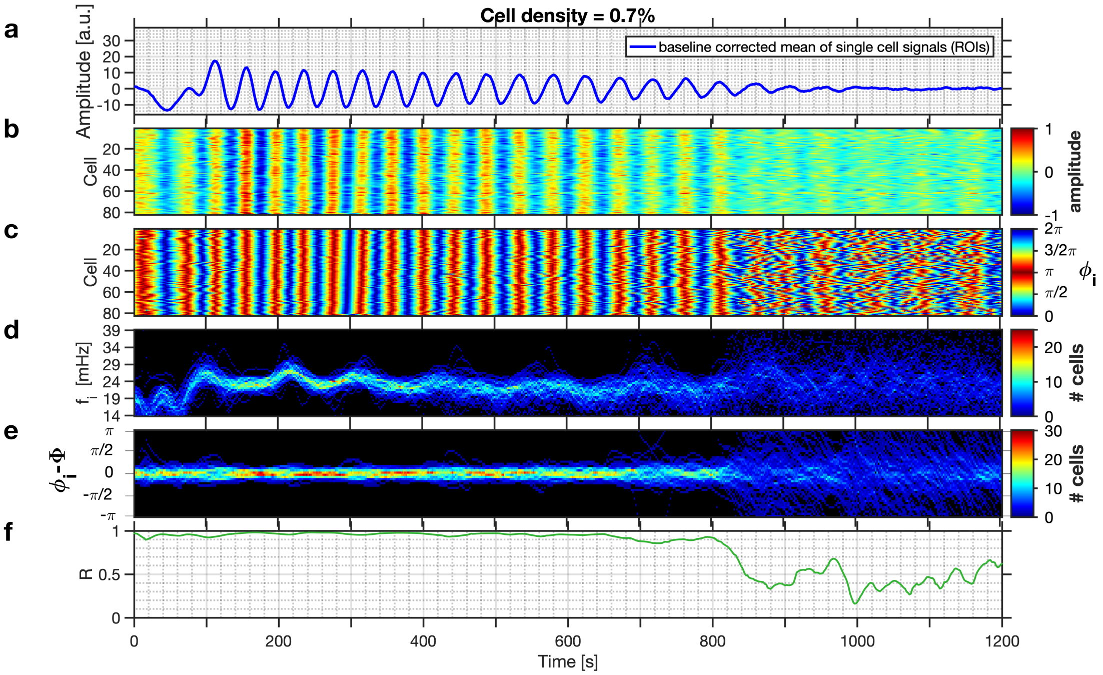

FIG. S11. (a) The time-series of the collective NADH fluorescence signal for a yeast population of high cell density, i.e., $\rho = 0.7\%$. Complete synchronisation of intercellular oscillations occurs almost immediately. (b) Development of the relative amplitudes of oscillations of each cell, and (c) of their phases. In (b) and (c) the cells are sorted according to their phases at time $t = 300\,\mathrm{s}$. (d) Evolution of the distribution of instantaneous frequencies f_i of the cells, and (e) of the distribution of the phase difference $\Delta\phi_i$ between the phase ϕ_i of each individual cell to that of the average phase Φ of all cells of the population. (f) Time dependence of the order parameter R. From the begin of the experiment, R reaches almost unity (up to $t \approx$ 660 s), indicating a complete synchronisation of the cells to a common rhythm. Glucose was added to the cell suspension at $t = -74\,\mathrm{s}$.

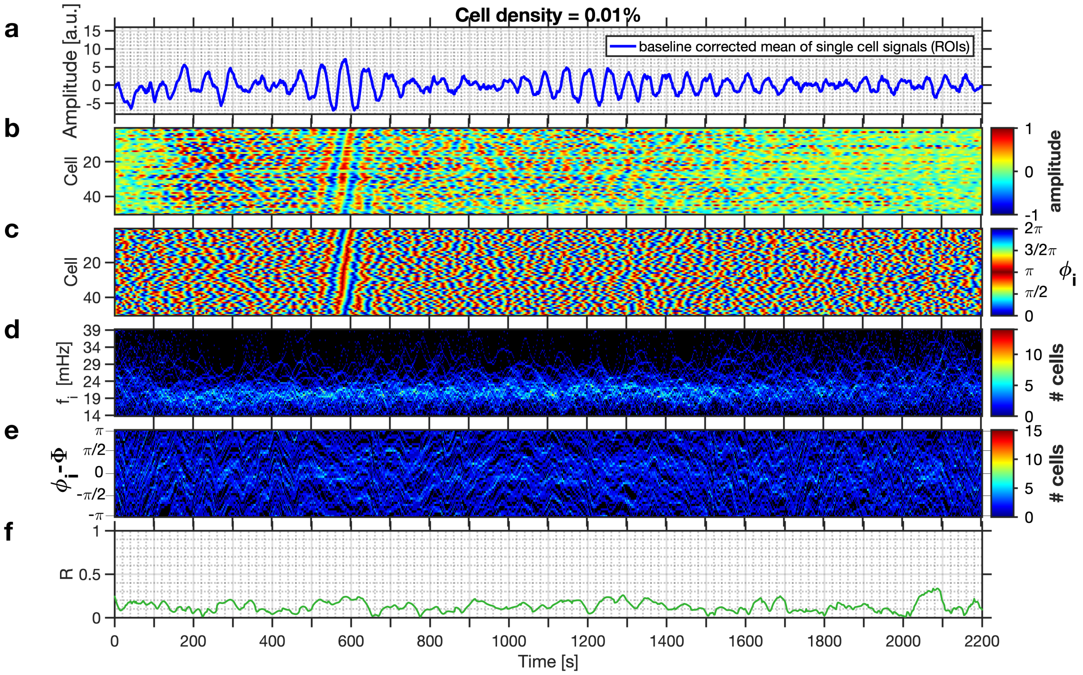

FIG. S12. (a) The time-series of the collective NADH fluorescence signal for a yeast population of low cell density, i.e., $\rho = 0.01\%$. Partial synchronisation of intercellular oscillations does not occur at all during the experiment. (b) Development of the relative amplitudes of oscillations of each cell, and (c) of their phases. In (b) and (c) the cells are sorted according to their phases at time $t = 600\,\mathrm{s}$. (d) Evolution of the distribution of instantaneous frequencies f_i of the cells, and (e) of the distribution of the phase difference $\Delta\phi_i$ between the phase ϕ_i of each individual cell to that of the average phase Φ of all cells of the population. (f) Time dependence of the order parameter R, which always remains below 0.3, indicating that partial synchronisation is never attained. Glucose was added to the cell suspension at $t = -150\,\mathrm{s}$.
